# Disruption of zinc finger DNA binding domain in catabolite repressor Mig1 increases growth rate, hyphal branching, and cellulase expression in hypercellulolytic fungus *Penicillium funiculosum* NCIM1228

**DOI:** 10.1186/s13068-018-1011-5

**Published:** 2018-01-25

**Authors:** Anmoldeep Randhawa, Olusola A. Ogunyewo, Danish Eqbal, Mayank Gupta, Syed Shams Yazdani

**Affiliations:** 1 Microbial Engineering Group, International Centre for Genetic Engineering and Biotechnology, Aruna Asaf Ali Marg, New Delhi, 110067 India; 2 DBT-ICGEB Centre for Advanced Bioenergy Research, International Centre for Genetic Engineering and Biotechnology, Aruna Asaf Ali Marg, New Delhi, India

**Keywords:** Carbon catabolite repression, Mig1 orthologs, Zinc finger domains, De-repression, Cellulases

## Abstract

**Background:**

There is an urgent requirement for second-generation bio-based industries for economical yet efficient enzymatic cocktail to convert diverse cellulosic biomass into fermentable sugars. In our previous study, secretome of *Penicillium funiculosum* NCIM1228 showed high commercial potential by exhibiting high biomass hydrolyzing efficiency. To develop NCIM1228 further as an industrial workhorse, one of the major genetic interventions needed is global deregulation of cellulolytic genes to achieve higher enzyme production. Mig1 orthologs found in all yeast and filamentous fungi are transcriptional regulators that maintain carbon homeostasis by negatively regulating genes of secondary carbon source utilization. Their disruption has long been known to be beneficial for increasing the production of secreted enzymes for alternate carbon source utilization.

**Results:**

Upon detailed genotypic and phenotypic analysis, we observed that NCIM1228 harbors a truncated yet functional allele of homolog of a well-known catabolite repressor, Mig1. Alleviation of carbon repression in NCIM1228 was attained by replacing functional Mig1^134^ allele with null allele Mig1^88^. *P. funiculosum* having Mig1^88^ null allele showed better growth characteristics and 1.75-fold better glucose utilization than parent strain. We also showed that visibly small colony size, one of the major characteristics of CCR disruptant strains in filamentous fungi, was not due to retarded growth, but altered hyphal morphology. CCR-disrupted strain PfMig1^88^ showed profuse branching pattern in terminal hyphae resulting in small and compact colonies with compromised filamentous proliferation. We further observed that basal level expression of two major classes of cellulases, namely, cellobiohydrolase and endoglucanase, was regulated by Mig1^134^ in NCIM1228, whereas other two major classes, namely, xylanases and β-glucosidase, were only marginally regulated. Finally, CCR disruption in *P. funiculosum* NCIM1228 led to prolonged cellulase induction in production medium resulting in twofold increased cellulase activity than the parent strain with maximum secreted protein titer being > 14 g/l.

**Conclusions:**

CCR-disrupted *P. funiculosum* showed better growth, enhanced carbon source utilization, profuse branching pattern in terminal hyphae, and higher cellulase activity than parent strain. Our findings are particularly important in shedding light on important functions performed by Mig1 in addition to its role as negative regulator of alternate carbon source utilization in filamentous fungi.

**Electronic supplementary material:**

The online version of this article (10.1186/s13068-018-1011-5) contains supplementary material, which is available to authorized users.

## Background

Efficient yet economic conversion of lignocellulosic biomass into metabolisable sugars continues to be a major bottleneck in the development of second-generation bio-based industries [1]. Filamentous fungi, being the major carbon recyclers of the earth, have inherent capacity to hydrolyze recalcitrant lignocellulose [2, 3]. Further, unprecedented ability of protein secretion makes them the preferred choice of bioprospecting for superior cellulolytic enzymes [4, 5]. Recent bioprospecting in our lab identified a strain of filamentous fungus *Penicillium funiculosum* (NCIM1228) having secretome of outstanding biomass hydrolyzing potential [6]. Proteomic studies of the NCIM1228 secretome revealed that Carbohydrate Active Enzymes (CAZymes) constitute ~ 58% of the total proteins secreted under cellulase inducing conditions [6]. Further genetic improvement of *P. funiculosum* is needed for enhanced production of lignocellulolytic cocktail for industrial use [4]. Two molecular approaches that have been used in the literature for achieving higher cellulolytic enzyme levels are (i) over-expression of key proteins involved and (ii) deregulating the expression of key enzymes. In *P. funiculosum* secretome, percentage levels of key rate limiting enzymes Cellobiohydrolase I (CBHI) and Cellobiohydrolase II (CBHII) in secretome were found to be relatively low as compared to *Trichoderma reesei* [6]. In this context, the study signified synergistic action of cellulolytic enzymes and non-hydrolyzing proteins found in the secretome in deconstructing the biomass [6]. Accordingly, over-expressing selected cellulolytic enzymes would not give the desired results as it can disturb the corresponding ratio of other accessory enzymes involved in the deconstruction process. Rather altering global regulatory mechanisms of cellulase induction would be beneficial in balanced increase in the expression of all cellulolytic enzymes [7].

Carbon catabolite repression (CCR) is a sophisticated control mechanism that regulates external and internal metabolism of the fungi depending upon the availability of carbon sources. It is part of global transcriptional regulatory mechanism which negatively regulates the expression of cellulolytic enzymes [7, 8]. The CCR is primarily mediated by transcriptional repressor proteins called catabolite repressors. Mig1 was identified as the first catabolite repressor in *S. cerevisiae* [9]. Functionality of *S. cerevisiae* Mig1 (ScMig1) is attributed to the presence of class I zinc finger domains found at its N-terminus [10, 11]. ScMig1 responds to abundant glucose availability by translocating to nucleus along with hexokinase 2 to form repressor complex and binds to promoters of genes involved in alternate carbon utilization [12]. Low levels of glucose lead Mig1 and hexokinase 2 to migrate back to cytoplasm releasing repression by proteolytic degradation. However, upon low glucose availability, Mig1 acts as positive regulator of filamentous MAPK pathway and favors filamentous growth to search for alternate carbon source [13]. Orthologs of Mig1 were later identified in cellulase producing filamentous fungi, namely, *Aspergillus niger*, *Neurospora crassa*, *Trichoderma reesei*, *Penicillium oxalicum*, and *Acremonium cellulolyticus* [14–21]. Fungal orthologs of Mig1 have highly conserved class I zinc finger (C2H2) domain, with moderate sequence similarity to ScMig1. CreA of *Aspergillus nidulans* (AnCreA) is the most studied homolog of Mig1 in filamentous fungi. Studies on AnCreA revealed the presence of additional domains which are also relatively conserved among filamentous fungi [21]. Here, zinc finger domains are followed by alanine-rich region which is believed to keep DNA binding zinc finger domains away from regulatory domain found at C-terminal end of AnCreA [21, 22]. Regulatory domain is preceded by acidic-rich region, which is believed to determine the On/Off state of CreA repressor based on its phosphorylation status. C-terminal regulatory domain of AnCreA has sequence very similar to Rgr1 (Subunit of RNA polymerase II mediator complex; required for glucose repression in *S. cerevisiae*) [14]. Studies on null alleles of AnCreA revealed that zinc finger domains are fundamental to its function, whereas other domains, though involved in repression, are dispensable [23, 24]. Alleles without C-terminal domains had impaired repression; however, DNA binding ability of CreA was found to be intact [23, 24]. Any disturbance in zinc finger domain either diminished or abolished the activity of AnCreA. Disruption of zinc finger domains of Cre1/CreA repressors resulted in increased cellulase expression in filamentous fungi [19, 25, 26]. Industrial strain of *Trichoderma reesei* RutC30 used for cellulase production was also found to have disrupted CreA protein. The truncation was found to occur between the two zinc finger domains [27].

In the present study, we attempted to study the catabolite repression in yet to be characterized *P. funiculosum* NCIM1228 and exploited it to produce high levels of cellulolytic enzymes for second-generation biofuel industries. We found PfMig1 to be among the evolutionary most evolved group across fungal kingdom. Sequence analysis identified PfMig1 gene of NCIM1228 to encode a truncated yet functional catabolite repressor Mig1^134^. By homologous recombination, we disrupted zinc finger domains of Mig1^134^ resulting in a null allele Mig1^88^. The resultant strain PfMig1^88^ was carbon catabolite de-repressed. Growth of PfMig1^88^ seemed compromised on solid media as reported by studies in Cre deletion mutants of other filamentous fungi; however, we found that PfMig1^88^ grew faster than parent strain in liquid media. Careful microscopic examination of colonies of NCIM1228 and mutant carrying null allele of Mig1 revealed that compact colony size in PfMig1^88^ was due to profuse branching which probably compromised the proliferating ability of hyphae on solid agar. Alleviation of catabolite repression led to increased basal expression level of cellulase transcripts and consequently enhanced secretion of major exo- and endo-cellulases.

## Results

### PfMig1 encodes a truncated but functional class I zinc finger domain transcription factor

**Fig. 1:**
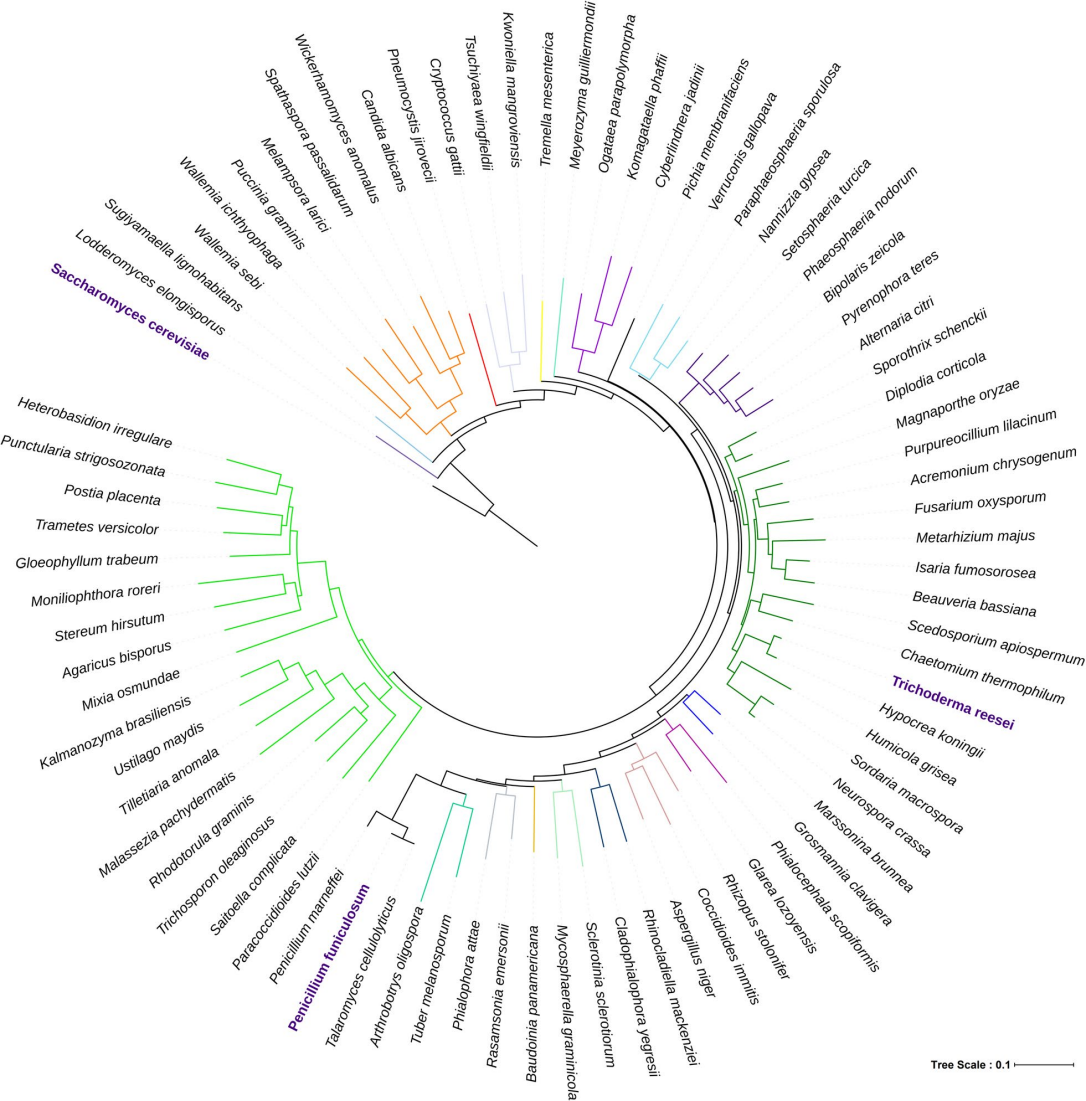
Phylogenetic tree of Mig1 orthologs in fungi. Mig1 nucleotide sequences of 41 industrially important fungal species representing 31 orders of fungal kingdom were taken to construct the phylogenetic tree. Mig1 sequence of *Saccharomyces cerevisiae* was used for rooting the Phylogenetic tree. PfMig1 shares one of the most recently evolved clades along with *Penicillium marneffei* and *Talaromyces cellulolyticus*

Since orthologs of Mig1 have been identified as CreA in fungi, we used CreA gene sequence of closest fungal species to *P. funiculosum* NCIM1228, i.e., *Talaromyces cellulolyticus* (dbj⎮BAO51847.1⎮), as query for blast search of in-house available draft genome database of *P. funiculosum.* We identified a putative class I zinc finger transcriptional repressor with 99% percentage identity and 100% coverage. Phylogenetic analysis was done based on nucleotide sequence of Mig1 orthologs of 194 fungal species (Additional file 1: Figure S1). To establish evolutionary relationship of *P. funiculosum* Mig1 (PfMig1), 41 species representing 31 orders across fungal kingdom were taken to construct phylogenetic tree (Fig. 1). Twenty-three major phylogenetic clades were identified where PfMig1 was found to constitute distinct clade from other cellulase producing fungi like *Trichoderma reesei* and *Aspergillus* sp. It shared the clade with other highly evolved fungi, such as *T. cellulolyticus* and *P. marneffei*, and represented more recent radiations of evolutionary conserved catabolite repressor Mig1 (Fig. 1). Putative Mig1 ortholog showed relatively high sequence homology to *A. nidulans* CreA (81.1% similarity, 72.5% identity, 6.3% gap) and moderate sequence homology to *Trichoderma reesei* (64.1% similarity, 51.6% identity) and *Neurospora crassa* (64.1% similarity, 52.7% identity). However, sequence alignment of region corresponding to zinc finger domains of PfMig1 with other filamentous fungi indicated this region to be highly conserved among ascomycetes (Additional file 1: Figure S2). The presence of transcription factor Mig1 in all taxa of kingdom fungi marks its conserved role in catabolite repression.

**Fig. 2:**
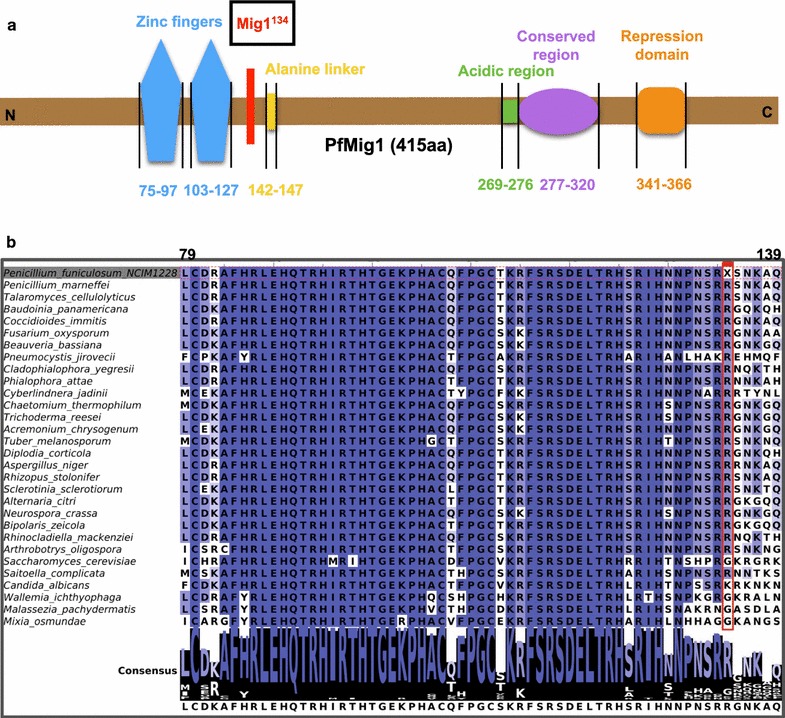
PfMig1 has a stop codon at 134th amino acid position. **a** Diagrammatic representation of putative domains detected in PfMig1. Red line states the presence of nonsense mutation at 134th amino acid position just before the alanine-rich region, thus naming the allele as Mig1^134^. **b** Sequence alignment of Mig1 protein of closely related fungi with PfMig1. The alignment indicates that the stop codon at 134th position was not due to natural selection, but was rather due to the result of laboratory induced mutation. Sequence from amino acid position 79–139 was taken as query. Red box indicates the 134th position corresponding to stop codon of PfMig1

The 2248-bp genomic region of NCIM1228 containing Mig1 ORF of 1248-bp along with 500-bp flanking region on both sides was amplified and cloned in pCambia1302 vector to obtain pCAR4a construct. The predicted protein was of 415 aa in length and contained two zinc finger domains (75–97 aa, 103–127 aa), an alanine-rich linker domain (142–147 aa), acidic activation domain (269–276 aa), and Rgr1-similar repression domain (341–366 aa) [28] (Fig. 2a). To our surprise, the annotation of Mig1 region in the draft genome sequence of NCIM1228 led to identification of a stop codon within the Mig1 ORF, leading to premature termination of translation at 134th amino acid position. We further confirmed the existence of stop codon within the Mig1 ORF of NCIM1228 by DNA sequencing of PfMig1 gene by Sanger method (Additional file 1: Figures S3, S4). To enquire whether there is a possibility of the presence of stop codon in Mig1 of other natural fungal isolates, nucleotide sequence alignment of PfMig1 with Mig1 gene from 194 fungal isolates, as named in Additional file 1: Figure S1, was performed. No other fungal isolates showed stop codon at the position where it was found in PfMig1, and therefore it is unlikely that this mutation was naturally acquired by NCIM1228. However, this strain was mutagenized in the earlier study to increase the cellulolytic enzyme expression [32]; thus, it is possible that the gene for Mig1, which has been shown to control the expression of various cellulolytic enzymes, has been mutated to partly relieve the repression. The alignment of Mig1 of 30 representative fungal isolates has been shown in Additional file 1: Figure S5. It appears from the alignment that opal stop codon in PfMig1 was a result of A/T transversion at 400th nucleotide position since its neighboring isolates have nucleotide ‘A’ at that position (Additional file 1: Figure S5). This has led to translation of a truncated protein of 133 aa in length in NCIM1228 instead of native Mig1 protein of 415 aa (Fig. 2b). The truncated protein would contain intact two zinc finger domains and would lack alanine-rich linker domain, acidic activation domain, and repression domain (Fig. 2a).

**Fig. 3:**
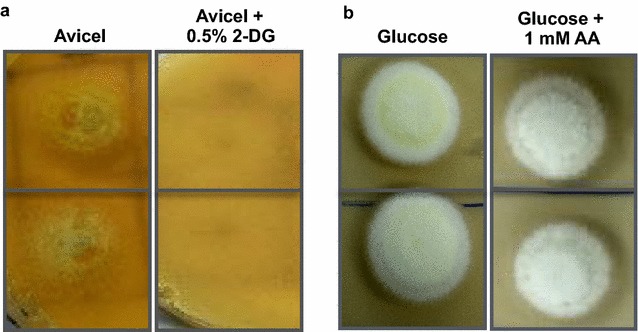
Mig1^134^ is a functional allele. **a** Conidiospores of NCIM1228 carrying Mig1^134^ allele was spotted in duplicate on SC media having 2% Avicel in the absence and presence of 0.5% 2-deoxyglucose (2-DG) and was found sensitive to it. **b** Conidiospores of NCIM1228 were spotted in duplicate on SC media having 2% glucose in the absence and presence of 1 mM allyl alcohol (AA) and was found resistant to it

The Cre mutant alleles of *Aspergillus niger* have earlier been characterized for the extent of carbon de-repression using 2-deoxyglucose and allyl alcohol [23]. To determine status of CCR in *P. funiculosum* NCIM1228 in the presence of truncated allele, we evaluated the resistance of the fungus to 2-deoxyglucose (2-DG) [29]. 2-DG is a non-metabolisable analogue of glucose which gets phosphorylated upon entering the cell and constitutively activates CCR. As a result, strains with functional CCR will not be able to grow on alternative carbon sources in the presence of 2-DG. However, strains with impaired CCR are insensitive to 2-DG presence and will grow normally on alternate carbon sources. When *P. funiculosum* NCIM 1228 was grown on 2% Avicel supplemented with 0.5% 2-DG, the strain was found to be sensitive to 0.5% 2-DG (Fig. 3a). This result suggested that CCR is functionally active in *P. funiculosum* NCIM1228. We also tested CCR functionality by using allyl alcohol (AA). Allyl alcohol is converted to a toxic compound acrolein by alcohol dehydrogenase, which would not allow the cells to grow. Upon abundant glucose availability, the functional CCR inhibits the expression of alcohol dehydrogenases, whereas the impaired CCR results in leaky expression of alcohol dehydrogenases thereby preventing the growth of the cells in the presence of AA. For this assay, NCIM1228 was grown in the presence of 1% glucose with or without 1 mM allyl alcohol. The strain was found to be resistant to allyl alcohol at 1 mM concentration in the presence of glucose (Fig. 3b). These phenotypic evaluations suggest that CCR is functionally active in *P. funiculosum* NCIM1228. As a consequence, regulation of cellulase expression under repressing conditions was appropriately maintained in NCIM1228 even in the presence of nonsense codon at 134th position of Mig1.

### Carbon catabolite repression diminishes in the presence of PfMig1^88^ having disrupted zinc finger domain

**Fig. 4:**
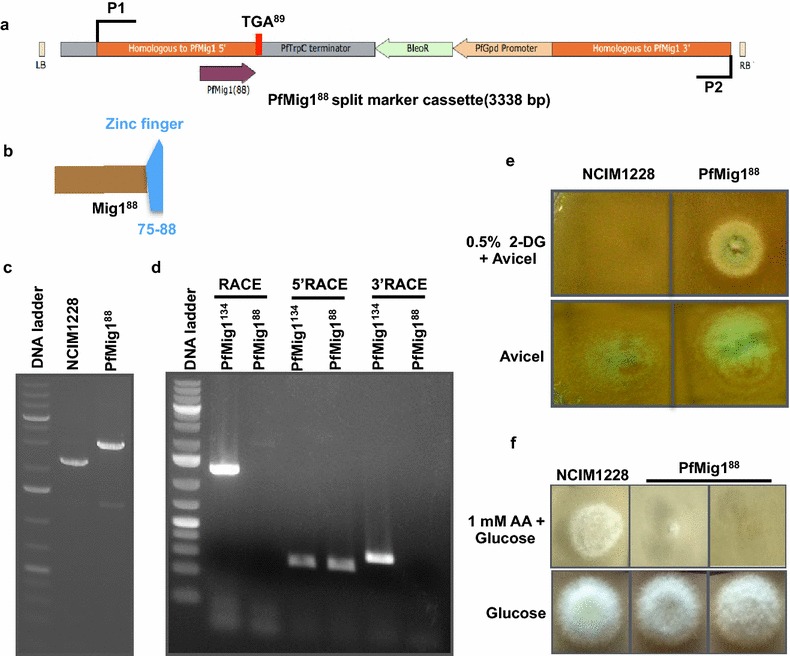
CCR is disrupted in PfMig1^88^. Graphical representation of **a** Mig1^88^ cassette constructed for homologous recombination and cloned in pCambia1302, and **b** Truncated Mig1^88^ protein. **c** PCR amplification products using Mig1 flanking primers. Lane 1 represents DNA ladder, Lane 2 shows amplification of 2248-bp fragment indicating native Mig1 gene and flanking sequence of NCIM1228, and Lane 3 shows amplification of 3035-bp fragment using P1 and P2 primers indicating homologous recombination of PfMig1^88^ cassette leading to loss of native Mig1 sequence. **d** RACE experiments to confirm the replacement of native gene with Mig1^88^ at RNA level. Lane 1 represents DNA ladder, Lane 2 shows amplification of 1248-bp band representing full-length Mig1 RNA in NCIM1228 and no amplification in Lane 3 shows its absence in PfMig1^88^ transformant, Lanes 4 and 5 represent 5′ RACE where 253-bp fragment from 5′ end of Mig1 RNA was amplified in both NCIM1228 and PfMig1^88^, and Lanes 6 and 7 represent 3′ RACE where NCIM1228 shows amplification of 319-bp fragment representing intact 3′-end of Mig1 RNA, whereas failed 3′ RACE in PfMig1^88^ shows truncated Mig1^88^ RNA. 1-kb Plus DNA ladder (Fermentas) was used as DNA marker. Conidiospores of NCIM1228 and PfMig1^88^ were spotted on **e** SC media having 2% Avicel in the absence and presence of 0.5% 2-DG, where PfMig1^88^ was found resistant to 2-DG, and on **f** SC media having 2% glucose in the absence and presence of 1 mM AA, where PfMig1^88^ was found sensitive to 1 mM AA

With intent to disrupt CCR in NCIM1228, a split marker disruption cassette was constructed in pCAR4a by replacing 264–903 bp in the ORF region of Mig1 with zeocin expression cassette (Fig. 4a). The resultant mutant allele, Mig1^88^, has stop codon inserted at 265 nucleotide position, followed by *trpC* transcription terminator from *P. funiculosum*. Hence, the mRNA transcribed by Mig1^88^ would be shorter in length (264 nucleotides) (Fig. 4b) than Mig1 which has intact mRNA of 1245 bp. The resultant plasmid pCMig1^88^ was used to transform *P. funiculosum* NCIM1228 by agrobacterium-mediated transformation method. Zeocin resistant transformants were screened by PCR (see “Methods” section for details) and transformants with amplified DNA product size consistent with Mig1^88^ split marker cassette were selected (Fig. 4c). The replacement was also confirmed by Rapid Amplification of cDNA Ends (RACE) using cDNA made from the transcripts of the NCIM1228 and the transformants having Mig1^88^ split marker cassette. RACE resulted in amplification of full-length mRNA in case of NCIM1228, while no amplification was observed for Mig1^88^ mutant indicating truncation of Mig1 gene here (Fig. 4d). When 200 bases each from 5′ and 3′ end of the Mig1 ORF were amplified using RACE, NCIM1228 showed amplification in both the cases, whereas PfMig1^88^ mutant showed amplification only for 5′ end of the ORF (Fig. 4d). This observation confirmed that mRNA had indeed been truncated in case of Mig1^88^ resulting in failed 3′-RACE, while it remained intact in case of Mig1^134^ (Fig. 4d).

Disruption of zinc fingers in Mig1 orthologs is known to cause defects in CCR. Therefore, the effect of Mig1^88^ mutant allele on CCR for NCIM1228 was examined by 2-DG and AA assay. When 2% Avicel was used as carbon source, strain having Mig1^88^ mutant allele (PfMig1^88^) was resistant to 0.5% 2-DG (Fig. 4e). It indicated that CCR was impaired in PfMig1^88^ strain. Defect in CCR as a consequence of zinc finger disruption in PfMig1^88^ was also confirmed by sensitivity of the strain carrying Mig1^88^ mutant allele to 1 mM allyl alcohol (Fig. 4f).

### PfMig1^88^ strain displayed better growth characteristics than NCIM1228

**Fig. 5:**
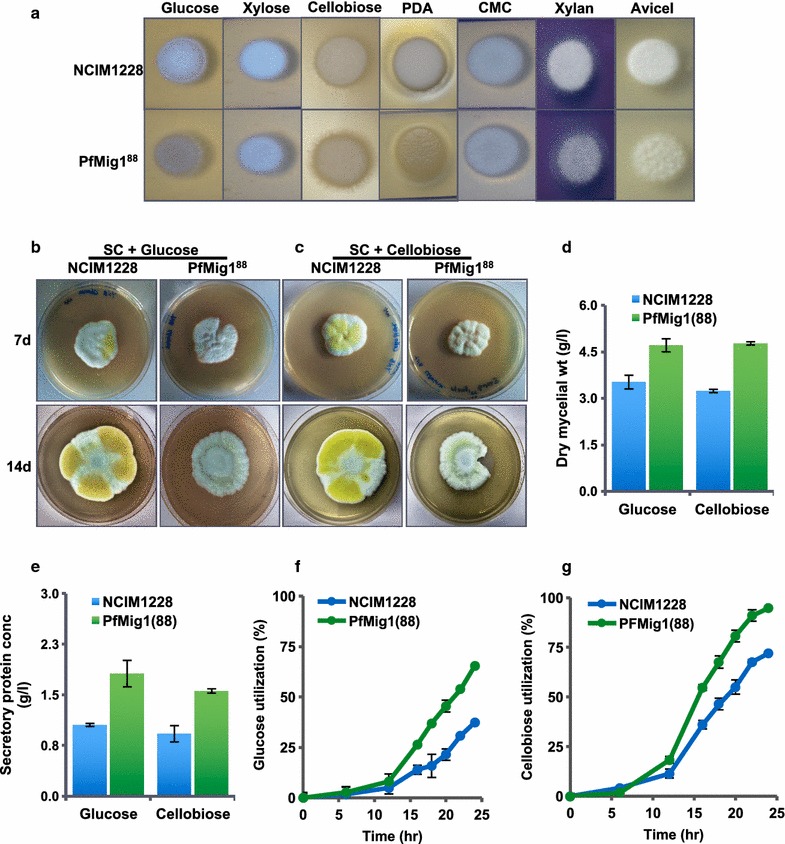
PfMig1^88^ features physiognomies different from NCIM1228. **a** 10 µl of 10^6^ conidiospore suspension of NCIM1228 and PfMig1^88^ were spotted on SC media having different carbon sources and growth of the cells was observed after 48 h. Appearance of colonies on **b** SC-glucose and **c** SC-cellobiose plate after 7 days and 14 days. **d** Mycelial biomass and **e** secretory protein concentration of PfMig1^88^ relative to NCIM1228 on SC-glucose and SC-cellobiose liquid media after 24 h of cultivation. Total glucose (**f**) and cellobiose (**g**) uptake rate when NCIM1228 and PfMig1^88^ were grown in SC liquid media having 1% glucose and 1% cellobiose, respectively. The uptake rate was monitored by measuring residual glucose and cellobiose remaining in the medium at various time intervals starting with equal spore count

**Fig. 6:**
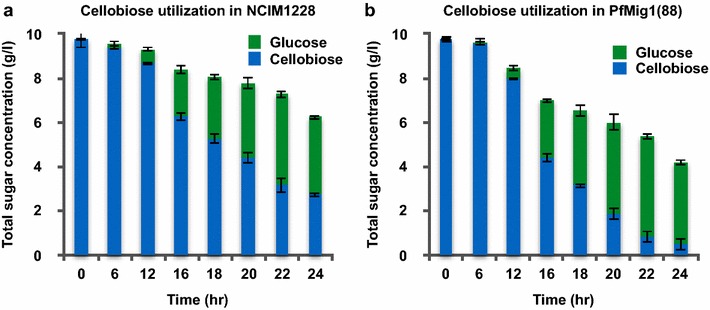
Cellobiose gets hydrolyzed to glucose before utilization. Residual cellobiose and glucose was monitored with respect to time for **a** NCIM1228 and **b** PfMig1^88^ strains grown in SC medium having 1% cellobiose

To investigate the influence of PfMig1^88^ mutant allele on the growth of NCIM1228, equal number of spores of NCIM1228 and PfMig1^88^ strain were spotted on SC agar plates having 2% carbon sources, namely, glucose, xylose, potato dextrose, xylan blue, cellobiose, carboxymethyl cellulose (CMC), and Avicel. PfMig1^88^ showed slightly weaker colony growth on most of carbon sources in 48 h (Fig. 5a). On SC agar plates having glucose and cellobiose, *P. funiculosum* NCIM1228 colonized as polarized hyphal growth at 28 °C and generally formed broadly spreading, white tufted colonies with smooth floccose and yellow pigmentation in 7–14 days (Fig. 5b, c). In contrast, PfMig1^88^ colonies were grayish and showed dissimilar morphology with rough floccose, reduced aerial hyphae, and grayish pigmentation. We also measured growth of NCIM1228 and PfMig1^88^ in the liquid SC medium having 1% glucose and 1% cellobiose as carbon source (Fig. 5d). In contrast to solid medium, there was 30% increase in the dry mycelial mass accumulated by PfMig1^88^ in 24 h. Quantification of secretome protein content of the cultures also exhibited substantial increase in the protein content of PfMig1^88^ mutant (Fig. 5e). We next measured the uptake of glucose in both the strains for different time periods ranging from 0 to 24 h. The uptake rate of glucose for PfMig1^88^ was 1.75-fold higher over the parent NCIM1228 at 24 h of growth (Fig. 5f). We also measured the utilization of cellobiose in both the strains. Cellobiose could be utilized either by its direct uptake or its extracellular hydrolysis to glucose by membrane-bound or secreted β-glucosidase in filamentous fungi. We thus measured the cellobiose and glucose left in the media over different periods of time at 10 g/l initial cellobiose concentration. We found that PfMig1^88^ was a better utilizer of cellobiose as compared to NCIM1228 (Fig. 5g). The cellobiose utilization in both strains seemed to be mainly via its hydrolysis to glucose (Fig. 6a, b). While NCIM1228 could utilize 70% of the cellobiose with total consumption (uptake as well as hydrolysis) of 7 g/l cellobiose in 24 h (Figs. 5g, 6a), the PfMig1^88^ strain completely utilized 10 g/l of the cellobiose during this period (Figs. 5g, 6b). We also observed increase in accumulation of glucose over the period of time in both NCIM1228 and PfMig1^88^ along with the utilization of cellobiose (Fig. 6a, b). It indicated that the rate of hydrolysis of cellobiose into glucose was higher than the rate of uptake of glucose. Nevertheless, significantly higher rate of cellobiose hydrolysis as well as glucose consumption in PfMig1^88^ indicated alleviation of carbon repression in this strain. This is the first report to our knowledge of any carbon de-repressed strain of filamentous fungi showing better growth and carbon utilization than the carbon repressed strain.

### PfMig1^88^ strain exhibited altered colony organization

**Fig. 7:**
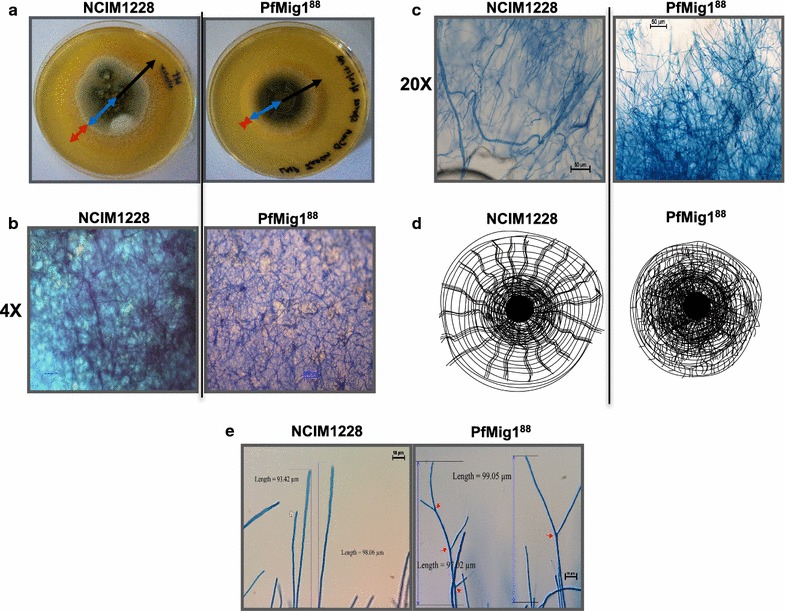
Presence of PfMig1^88^ allele induces profuse hyphal branching. **a** Colony morphology of NCIM1228 and PfMig1^88^ after 10 days of incubation. Black arrow denotes the radius of the whole colony, blue arrow denotes the extent of core and middle layer, and red arrow shows highly extended invading layer in NCIM1228 and highly reduced outer layer in PfMig1^88^. The outer layer of NCIM1228 and PfMig1^88^ cells were observed under fluorescence microscope at ×4 magnification (**b**) and at ×20 magnification (**c**). In NCIM1228, parallel grouped hyphal structures are shown running throughout the outer layer, whereas no such structures were observed in PfMig1^88^. **d** Graphical representation of colony morphology observed in NCIM1228 and PfMig1^88^. NCIM1228 colonies are organized around parallel hyphae running from core of the colony toward periphery, whereas no such impeded organization was observed in PfMig1^88^. **e** Branching pattern observed in terminal hyphae of NCIM1228 and PfMig1^88^ colonies

Since hyphal colonies of NCIM1228 do not easily mature and sporulate on SC agar, we used LMP agar (see “Methods” for details) to study colony structure of the two strains. LMP media permit sparse hyphal density with normal sporulation which enables easy observation of colony organization under microscope. When single spore of NCIM1228 was plated on LMP agar plate, it developed into a mature colony of diameter 68–75 mm in 10 days (Fig. 7a). The colonies of NCIM1228 displayed three well-grown mycelial layers with inner core, middle branched nodal mycelia and outer layer of invading mycelia of 12 mm in radius. In contrast, the colonies of PfMig1^88^ strain appeared more compact and restricted with a diameter of 38–40 mm with reduced aerial growth (Fig. 7a). Out of the three layers of colony, outermost layer of invading hyphae was the most affected in PfMig1^88^ having reduced width of 1–2 mm. For microscopic examination, mature colonies were stained with lactophenol blue and transverse sections of the colonies were observed under microscope. We identified stark difference in colony organization of the two strains. In case of NCIM1228, the colonies were organized into neat parallel bundles of unbranched hyphae which radiated out from the core and ended at the periphery of the colony (Fig. 7b–d). On the other hand, the PfMig1^88^ colonies showed incongruous growth with shorter and fewer bundles of parallel hyphae which ended midway of the colony. The colonies showed disorganized growth of highly branched and short hyphae which probably gave roughness to the colony surface (Fig. 7b–d). We next determined the branching pattern of terminal hyphae of the two strains. For this, up to 140 and 100 µm terminal hyphal lengths of NCIM1228 and PfMig1^88^, respectively, were considered. We found 70% of the total hyphae counted in NCIM1228 were unbranched, whereas only 8.8% of the hyphae of PfMig1^88^ were unbranched (Fig. 7e and Table 1). Most of the branching in NCIM1228 was in the form of nicked bifurcation where the branch did not grow into complete hyphae, whereas 50% of the terminal hyphae in PfMig1^88^ showed bifurcated branching where both the branches grew equally well. More than two branches were found in 30% of the terminal hyphae in PfMig1^88^, whereas it was a rare sight in NCIM1228 (Table 1). This showed that hyphal development got affected upon deletion of PfMig1. The absence of Mig1 led to branching in terminal hyphae, thereby hindering their invading capacity. However, it might enhance enzyme secretion as secretion occurs only at the hyphal tip and more hyphal tips might lead to more enzyme secretion.

**Table 1:** Percentage of hyphae showing distinct morphologies in *P. funiculosum* NCIM1228 and PfMig1^88^

% pattern in hyphal termini^a^	% unbranched	% nicked bifurcation	% bifurcation	% trifurcation	% quadra-furcation
NCIM1228	72.6 ± 4.4	16.5 ± 0.2	6.07 ± 3.5	4.76 ± 0.7	0
PfMig1^88^	8.8 ± 0.9	0	50 ± 0.47	30.5 ± 0.2	11 ± 1.6

^a^Branching pattern has been schematically represented in Additional file 1: Figure S6

### Effect of PfMig1^88^ null allele on sporulation

**Fig. 8:**
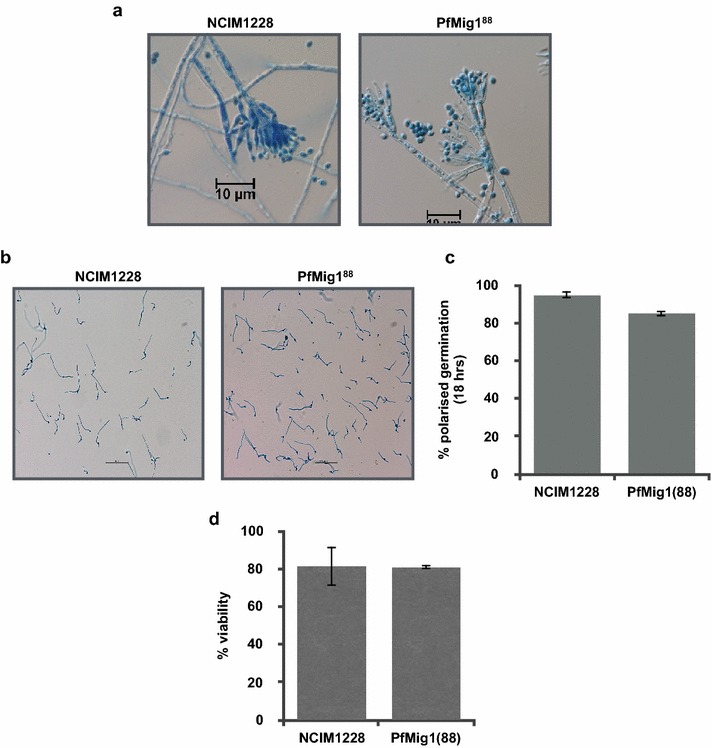
Asexual life cycle is not affected in the presence of Mig1^88^ allele. **a** Microscopic evaluation of (**b**) conidiophores at ×60 magnification and **b** germinating spores at ×20 magnification in NCIM1228 and PfMig1^88^. **c** Percentage germination of spores for NCIM1228 and PfMig1^88^ after 18 h of growth in PD broth. **d** Percentage viability of NCIM1228 and PfMig1^88^ calculated by plating 50 spores of each strain on PD plates and counting the colonies appeared after 72 h

Upon maturation, vegetative hyphae of *P. funiculosum* produce specialized structures called conidiophores that constitutes stalk on which metulae appear. Metulae further give rise to phialides on which uninucleate conidia are sequentially formed in budding manner, which are capable of forming new colonies. The conidiophores produced by NCIM1228 and PfMig1^88^ strains were observed under fluorescence microscope after staining with lactophenol blue (Fig. 8a). We did not find any significant change in the conidiophore structure and appearance of the two strains (Fig. 8a). Upon colony maturation, sporulation started from the core and slowly spread to all layers in NCIM1228, whereas sporulation in PfMig1^88^ was delayed and restricted to core of the colony. To quantify any possible increase or decrease in the conidiation, conidiospore density was calculated by spreading 10^4^ spores of both the strains on sporulating medium containing agar plate and calculating the conidiospore density in the colony after 10 days. PfMig1^88^ strain (1.32 × 10^6^ spores/mm^2^) showed only marginal difference in colonial density when compared with NCIM1228 (1.43 × 10^6^ spores/mm^2^) (Table 2).

**Table 2:** Spore density observed in *P. funiculosum* NCIM1228 and PfMig1^88^

Strains	NCIM1228	PfMig1^88^
Spore density (spores/mm^2^)	(1.43 ± 0.44) * 10^6^	(1.32 ± 0.08) * 10^6^

Integrity of the asexual conidiospores was determined for PfMig1^88^ strain in comparison to NCIM1228. Upon arrival of favorable conditions, dormant conidia germinate by initiating isotropic growth extending into highly polarized germ tubes (Fig. 8b). In order to examine if the conidia produced by the PfMig1^88^ strain exhibit any defect in germination, germination rate was measured by incubating conidia from both the strains in PD broth at 28 °C. In a population of 100, germinating and non-germinating spores of both the strains were counted in three independent experimental replicates (Fig. 8c). More than 80% of spores from both the strains could germinate into polarized germ tubes in 18 h of incubation. Even the viability tests of spores for PfMig1^88^ strain showed indistinct viability from the parental control (Fig. 8d). This suggested that the deletion of Mig1 in NCIM1228 did not cause any defect in asexual life cycle of *P. funiculosum*.

### Effect of impaired CCR on expression of cellulases and hemicellulases in *P. funiculosum* NCIM1228

**Fig. 9:**
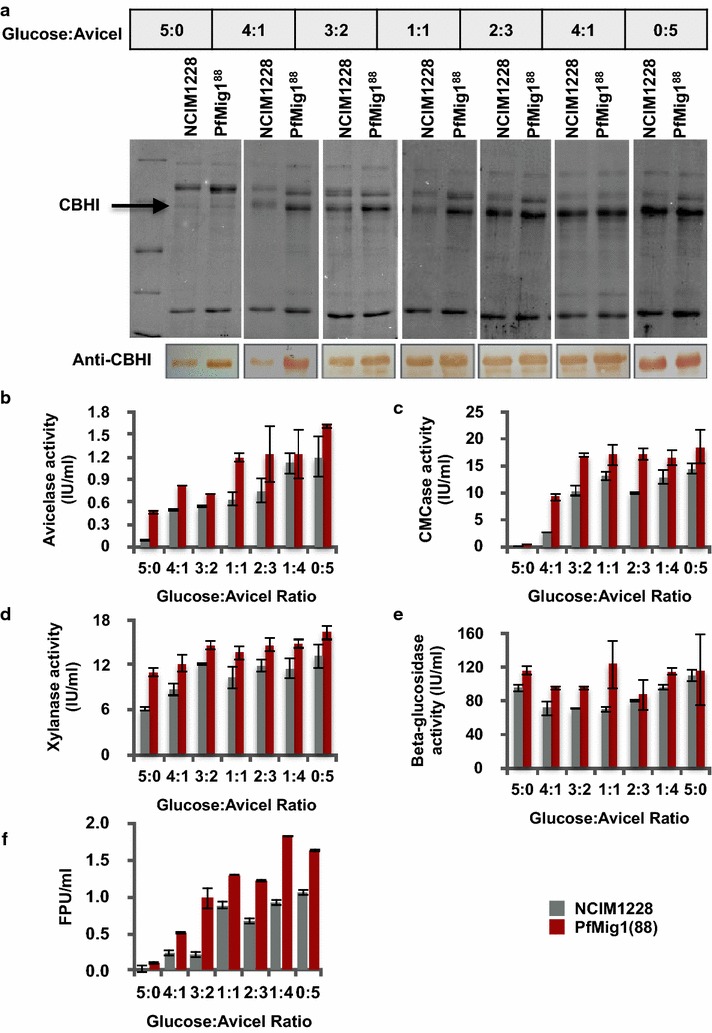
Secretome profiling of NCIM1228 and PfMig1^88^ in the presence of glucose. **a** Upper panel shows SDS-PAGE profile of 10 times diluted secretome of NCIM1228 and PfMig1^88^ grown in Mandel media having varying ratios of glucose and Avicel. Lower panel shows detection of CBHI under different conditions by Western blotting using rabbit anti-CBHI primary antibody and HRPO-conjugated anti-rabbit secondary antibody. Activities for **b** Avicelase, **c** CMCase, **d** xylanase, **e** β-glucosidase, and **f** FPase were measured in the secretome of NCIM1228 and PfMig1^88^ when grown under different ratios of glucose and Avicel for 5 days

Expression of cellulases and hemicellulases is known to be negatively regulated by catabolite repressors. The presence of cellulosic carbon sources along with depleting glucose concentration in the media would induce their expression. Impaired CCR in the PfMig1^88^ strain might induce the expression of cellulases even in the presence of glucose. To examine this, we cultured both NCIM1228 and PfMig1^88^ strains under varying concentrations of glucose and Avicel while maintaining total carbon source concentration at 5%. Total secretory proteins of the cultures were analyzed on SDS-PAGE gel by loading equal volume of secretomes. We found increased secretome concentration in PfMig1^88^ strain under all media conditions (Fig. 9a). The differences were prominent when glucose and Avicel were present in the ratio of 4:1, 3:2, and 1:1. Increased concentration of secretome was also found under complete repressing (5% glucose) and de-repressing (5% Avicel) conditions. Cellobiohydrolase I (CBHI) is the most important exocellulase produced by NCIM1228 for crystalline cellulose breakdown [6, 30]. CBHI is also the most dominant cellulase found in the secretome of NCIM1228 [6, 30]. We thus evaluated the amount of CBHI being expressed under each condition by Western blotting using anti-PfCBHI antibodies. Relatively high levels of CBHI were detected in PfMig1^88^ strain under repressing as well as de-repressing conditions (Fig. 9a). Remarkable difference was found, when glucose and Avicel were added in the media in the ratio of 5:0, 4:1, 3:2, and 1:1 (Fig. 9a).

Secretomes of NCIM1228 and PfMig1^88^ strains grown under different ratios of glucose and Avicel were used for measuring activities of four major cellulolytic enzymes, i.e., exocellulase (by measuring Avicelase activity), endocellulase (by measuring CMCase activity), β-glucosidase (by measuring PNPG activity), and xylanase (by measuring activity against xylan). In addition, synergistic action of all cellulases was also determined by Filter Paper Unit (FPU) Assay. The activities were measured by incubating secretomes with specific substrates at 50 ºC (see Methods for details). We found that PfMig1^88^ strain outperformed the parent strain under all growth conditions for Avicelase, CMCase, and FPU activities (Fig. 9b, c, f). Under complete repressing condition of 5% glucose (glucose:Avicel ratio being 5:0), Avicelase and CMCase were found to be 0.09 IU/ml and 0.08 IU/ml in NCIM1228 secretome as against 0.5 and 0.4 IU/ml in PfMig1^88^ secretome, respectively (Fig. 9b, c), indicating more than seven- and fivefold increase in Avicelase and CMCase activity, respectively, in PfMig1^88^ strain. Only moderate increase was observed in xylanase and β-glucosidase activities of PfMig1^88^ secretome in comparison to NCIM1228 secretome (Fig. 9d, e). The above observation showed that exocellulase and endocellulase activities were particularly increased under alleviated carbon repression in PfMig1^88^ strain, whereas xylanase and β-glucosidase activities were moderately increased. The FPU activity was also found to be fivefold higher in case of PfMig1^88^ as compared to NCIM1228 under complete repressing condition, indicating overall increase in cellulase expression for PfMig1^88^ strain. Under complete de-repressing conditions (glucose:Avicel ratio being 0:5), we found 1.6-fold higher FPU activity in PfMig1^88^ when compared to NCIM1228. Highest FPU of 2.0 was achieved in PfMig1^88^ when glucose and Avicel were provided in the ratio of 4:1, which was twofold higher than NCIM1228. From these observations, we could conclude that increased exocellulase and endocellulase expression in the absence of functional Mig1 led to overall increase in synergistic action of all cellulases.

**Fig. 10:**
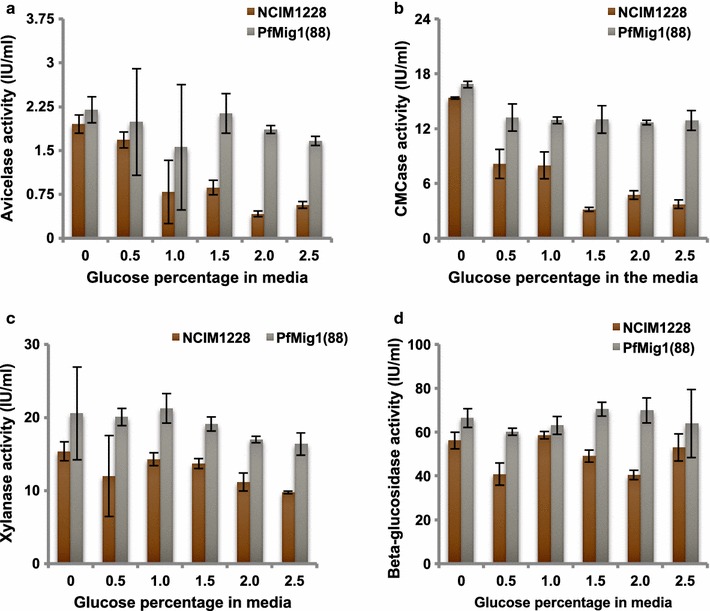
Assessing inhibiting concentration of glucose in the presence of inducer for NCIM1228 and PfMig1^88^. Activities for **a** avicelase, **b** CMCase, **c** xylanase, and **d** β-glucosidase were measured in the secretome of NCIM1228 and PfMig1^88^ when grown under 2% Avicel and glucose concentration ranging from 0 to 2.5%

Further, we determined the inhibiting concentration of glucose for NCIM1228 and PfMig1^88^ strains in the presence of fixed inducer concentration. For this, we grew the strains in the presence of 2% Avicel (inducer) with varying concentrations of glucose (repressor) at 0, 0.5, 1, 1.5, 2, and 2.5%. The secretomes collected were used for measuring activities of major classes of cellulases (Fig. 10a–d). In case of NCIM1228, exocellulase activity reduced by 40, 50, 80, and 90% in the presence of 1, 1.5, 2, and 2.5% glucose, respectively, as compared to the condition in which no glucose was added (Fig. 10a). On the other hand, exocellulase activity showed marginal decrease at high glucose concentration in case of PfMig1^88^ strain. We found similar pattern in endoglucanase activity of the NCIM1228 and PfMig1^88^ secretome under increasing concentration of repressing sugar (Fig. 10b). The presence of glucose as repressor had lesser impact on the activity of xylanase and almost no impact on the activity of β-glucosidase in the secretomes of both NCIM1228 and PfMig1^88^ strains (Fig. 10c, d). These observations indicated that expression of exocellulase and endocellulase was under the control of PfMig1 in NCIM1228, and that this control was relieved in the PfMig1^88^ mutant. On the other hand, expression of xylanase and β-glucosidase was least regulated by PfMig1, leading to no significant change in activity in both NCIM1228 and PfMig1^88^ strains when repressor (glucose) concentration was increased in the media.

### Increased transcript levels of cellulases in PfMig1^88^ strain

**Fig. 11:**
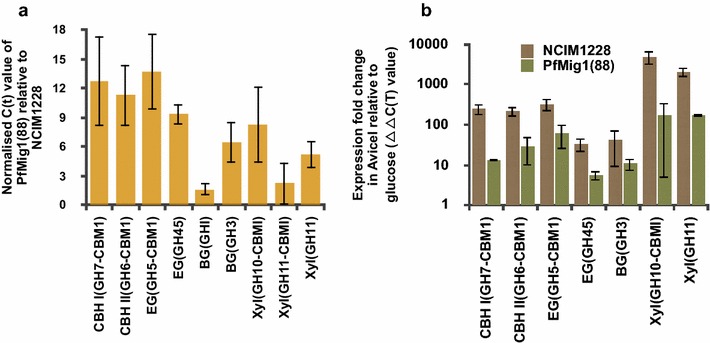
Cellulase expression gets de-repressed in PfMig1^88^. **a** The transcriptional expression of various families of cellobiohydrolase, endoglucanase, β-glucosidase, and xylanase in PfMig1^88^ were measured by quantitative real-time PCR after growing for 24 h in the presence of 4% glucose. The expression levels were normalized to NCIM1228 and plotted. **b** Transcript levels of various families of cellobiohydrolase, endoglucanase, β-glucosidase, and xylanase in NCIM1228 and PfMig1^88^ in the presence of 4% Avicel. Expression levels are shown in log scale and were normalized to NCIM1228 and PfMig1^88^ grown in the presence of 4% glucose

Increased cellulase activity in carbon-derepressed strain PfMig1^88^ might be due to deregulated expression of cellulases in the presence of glucose. We verified it by measuring transcript levels of some of the major cellulases of NCIM1228 and PfMig1^88^ in the presence of repressing and de-repressing conditions (Table 3). These cellulases are found in major amounts in NCIM1228 secretome under cellulase inducing conditions [6]. For this, NCIM1228 and PfMig1^88^ strains were grown for 24 h in the presence of 4% glucose (repressing condition) and 48 h (to achieve similar mycelial growth) in the presence of 4% Avicel (de-repressing condition). Transcript levels of the two strains under repressing and de-repressing conditions were determined by real-time PCR with tubulin as control. Under repressing conditions, there was 12- and 18-fold increase in the transcript levels of Cellobiohydrolase I (CBHI) and Cellobiohydrolase II (CBHII), respectively, in PfMig1^88^ relative to NCIM1228 (Fig. 11a). Similarly, we found more than 13- and ninefold increase in the transcript levels of endoglucanases (EG) of GH5 and GH45 family, respectively, in PfMig1^88^ strain in the presence of glucose. Out of the two β-glucosidases (BG) generally found in the NCIM1228 secretome under cellulose inducing conditions, i.e., BG(GH1) and BG(GH3), there was no difference found in the transcript levels of BG(GHI) in both the strains under repressing conditions, whereas transcript level of BG(GH3) was increased by sixfold in PfMig1^88^ (Fig. 11a). We determined transcript levels of three xylanases, namely, Xyl(GH10-CBMI), Xyl(GH11-CBMI), and Xyl(GH11) in NCIM1228 and PfMig1^88^. We observed eight- and fivefold increased Xyl(GH10-CBMI) and Xyl(GH11) transcript levels in PfMig1^88^ under repressing conditions, respectively. However, there was no difference found in transcript levels of Xyl(GH11-CBMI) between NCIM1228 and PfMig1^88^ under repressing conditions.

**Table 3:** List of CAZymes whose transcript levels were monitored in *P. funiculosum* NCIM1228 and PfMig1^88^

Enzyme class	Functional classification	Predicted CAZY family
Exocellulase	Cellobiohydrolase I (CBHI)	GH7-CBM1
	Cellobiohydrolase II (CBHII)	GH6-CBM1
Endoglucanase	Endoglucanase (EG)	GH5-CBM1
	Endoglucanase (EG)	GH45
Xylanase	Beta-1,4-xylanase (Xyl)	GH10-CBM1
	Beta-1,4-xylanase (Xyl)	GH11
	Beta-1,4-xylanase (Xyl)	GH11-CBM1
β-Glucosidase	Beta-glucosidase (BG)	GH1
	Beta-glucosidase (BG)	GH3

When transcript levels under de-repressing condition were compared to repressing condition, we found 250- and 217-fold increase in CBHI and CBHII transcripts in NCIM1228 in the presence of Avicel (Fig. 11b). However, only 13- and 29-fold increase was found in case of PfMig1^88^, respectively, under de-repressing vs repressing conditions. It shows that transcript levels under repressing conditions have indeed been increased in PfMig1^88^ leading to reduced fold change in transcript levels of major exocellulases in cellulose inducing conditions. We observed similar case with endoglucanases EG(GH5) and EG(GH45). EG(GH5) was found to have 331-fold increased transcript level in NCIM1228, whereas 61-fold increase was found for PfMig1^88^ in the presence of Avicel (Fig. 11b). The transcript for EG(GH45) was up-regulated by 34-fold in NCIM1228; however, fold change of transcript levels between repressing and inducing conditions was reduced to fivefold for EG(GH45) in PfMig1^88^. There was no up-regulation of BG(GH1) in de-repressing conditions in both NCIM1228 and PfMig1^88^ (data not shown); however, BG(GH3) showed up-regulation under de-repressing conditions in both the strains to almost similar extent (Fig. 11b). The fold change of Xyl(GH10-CBMI) and Xyl(GH11) was also lower for PfMig1^88^ than NCIM1228 in de-repressing condition. We did not find any change in the extent of up-regulation of Xyl(GH11-CBMI) under de-repressing conditions in both NCIM1228 and PfMig1^88^. Together with enzymatic activity assays, this experimental data suggested that major exocellulases and endocellulases were indeed being repressed by Mig1 in the presence of abundant glucose. The absence of Mig1 led to increased levels of major exoglucanases (CBHI and CBHII) and endoglucanases (EG-GH5 and EG-GH45) in secretome at basal level.

ScMig1 is known to bind to consensus sequence GCGGGG upstream to the genes of alternate carbon utilization [11]. Later, studies on Mig1 orthologs of filamentous fungi *A. nidulans* and *T. reesei* also indicated its binding at GCGGGG consensus sequence [18, 22]. Studies also revealed redundancy occurring at three of the positions of the consensus GCGGGG in the form of SYGGRG where S can be either C or G, Y can be either C or T, and R can be A or G. We checked for these consensus sequences upstream to CBHI, CBHII, EG(GH5), and EG(GH45) genes that were found to be de-repressed in PfMig1^88^ mutant strain. We indeed found the presence of putative Mig1 binding consensus sequence in all of them (Table 4). Both CBHI and CBHII were found to have four binding sites each between − 300 and − 1300 bp upstream to their ORFs. Endoglucanases GH5 and GH45 have 4 and 3 Mig1 binding sites, respectively, between − 50 and − 1200 bp upstream to their ORFs.

**Table 4:** Presence of putative Mig1 binding sites upstream of genes for major exocellulases (CBH) and endoglucanases (EG) in *P. funiculosum* NCIM1228

Gene/CAZY family name	Presence of consensus sequence in GCGGGG upstream to gene ORF	Presence of consensus sequence in XRGGPG upstream to gene ORF	Total number of putative PfMig1 binding site
*CbhI*/GH7	− 763 to − 769	− 523 to − 528	4
		− 639 to − 644	
		− 828 to − 833	
*CbhII*/GH6	− 1245 to − 1245	− 321 to − 326	4
		− 503 to − 508	
		− 1116 to − 1121	
*EG*/GH5	− 1066 to − 1071	− 56 to − 61	4
		− 506 to − 511	
		− 587 to − 592	
*EG*/GH45	− 159 to − 164	− 1200 to − 1205	3
		− 145 to − 150	

### Performance of PfMig1^88^ strain as cellulase producer

**Fig. 12:**
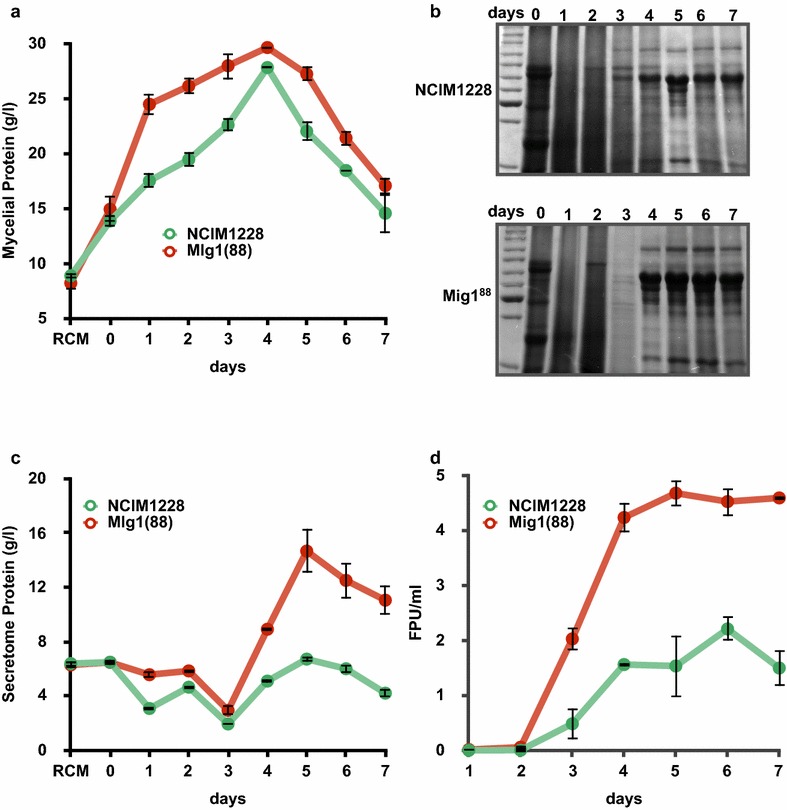
Enhanced cellulase secretion and activity was detected in PfMig1^88^ in production medium. **a** Growth of the fungus in production media was monitored for 7 days by estimating the mycelial proteins by BCA method. RCM in X-axis denotes the amount of protein present in the pellet of medium before inoculation. **b** Proteins present in the secretome of NCIM1228 (upper panel) and PfMig1^88^ (lower panel) were collected for each day and separated on 10% SDS-PAGE gel followed by staining with Coomassie blue. **c** Secretome proteins of NCIM1228 and PfMig1^88^ collected for each day was measured by BCA method after exchanging growth medium with citrate–phosphate buffer (pH 4.0). RCM in X-axis denotes the amount of protein present in the supernatant of medium before inoculation. **d** Total cellulase activity profile represented by FPU/ml of NCIM1228 and PfMig1^88^ during 7 days of cultivation

An ideal industrial workhorse for cellulase production will have superior secretome along with high secretion capabilities. We next examined if Mig1^88^ mutant allele would help in improving the economics of cellulase production by NCIM1228 in RCM production medium. RCM medium consists of cellulose and hemicellulose sources for inducing both cellulases and hemicellulases along with complex protein source to support fast growth. We analyzed growth characteristics and secretome of PfMig1^88^ and NCIM1228 in RCM throughout the fermentation. Since RCM medium is composed of high quantity of insoluble components making accurate determination of biomass difficult, growth of NCIM1228 and PfMig1^88^ was measured by estimating mycelial protein by BCA. PfMig1^88^ was found to grow faster than NCIM1228 in RCM medium; however, both strains attained maximum growth on 4th day of fermentation (Fig. 12a). Decrease in growth after 4th day was observed, possibly due to culture maturation and sporulation. Total protein content of NCIM1228 and PfMig1^88^ secretome was analyzed throughout the fermentation on SDS-PAGE gel as well as by BCA. Both the secretomes showed high protein content during the first 3 days; these proteins possibly represent the soluble soya proteins provided in the RCM medium (Fig. 12b). Decrease in protein content on 3rd day indicated consumption of soluble complex protein source by the mycelia. The secretome content was then found to increase on 4th day for both NCIM1228 and PfMig1^88^ strains. Maximum protein secretion was found on 5th day for both strains, though the magnitude of protein content of PfMig1^88^ was more than twofold higher than NCIM1228 reaching to > 14 g/l (Fig. 12c). Total cellulase activity of the secretomes was measured by Filter paper unit (FPU) assay (Fig. 12d). The maximum cellulase activity was found on 5th day for PfMig1^88^ and 6th day for NCIM1228. The secretome of PfMig1^88^ demonstrated maximum of 4.7 FPU/ml of cellulase activity, which was twofold higher than that of the NCIM1228 secretome (Fig. 12d).

## Discussion

An ideal industrial cellulase producing strain would have (1) high-performing secretome with balanced ratio of all enzymes needed for biomass hydrolysis, and (2) capability to grow fast and produce high amounts of cellulolytic enzymes in the presence of glucose. Fungal cellulases are under inducible expression and are greatly affected by CCR. To make the strain industrially viable, alleviation of carbon catabolite repression (CCR) becomes essential to achieve high level of cellulolytic enzymes in an industrial setup. A recent bioprospecting for high-performing secretome for saccharification resulted in identification of *P. funiculosum* NCIM1228 strain [6, 30]. NCIM1228 was earlier isolated based on its high cellulolytic activity after UV and chemical treatment of *P. funiculosum* [31, 32]. In this study, we attempted to further increase the cellulase production in NCIM1228 strain by disrupting the major catabolite repressor Mig1 found in all yeast and filamentous fungi. Phylogenetic analysis of Mig1 orthologs was done which revealed that PfMig1 belonged to one of the most recent radiations of Mig1 and may have attained additional roles. We identified a truncated PfMig1 allele having a A/T transversion mutation at 400th nucleotide resulting in stop codon at amino acid position 134 in *P. funiculosum* NCIM1228. Since this nonsense mutation was not observed in Mig1 ORF of any other natural fungal isolate, it could have been introduced during the mutagenesis process performed earlier on this strain to improve its cellulolytic activity [32]. This mutation would result in truncated Mig1 protein of ~ 14 kD having intact zinc finger domains and terminating just before alanine-rich region. Yet, NCIM1228 was found to have functional CCR as evident by its phenotypic response to 0.5% deoxyglucose and 1 mM allyl alcohol. This indicated that Mig1^134^ was functional keeping expression of cellulases and alcohol dehydrogenases under check in repressing conditions. The earlier reports on truncated alleles of *A. nidulans* CreA, i.e., *CreA30* and *CreA305* alleles, which had intact zinc finger domains and truncation just before alanine-rich regions, also indicated retaining of their DNA binding activity in the absence of other domains [23, 24]. This suggests that zinc fingers are the essential functional entities needed for repression and all the other domains have accessory role in carbon repression.

### Hyphal morphology, not growth, is affected in CCR-disrupted PfMig1^88^ strain

We replaced PfMig1^134^ allele in NCIM1228 with PfMig1^88^ allele by homologous recombination. Disruption cassette for Mig1 was made such that the functional entity of Mig1, i.e., zinc fingers, should get disrupted, but leaving flanking regions of Mig1 intact. This was done to avoid any phenotype that might emerge due to change in flanking region as shown in *Aspergillus niger* where disruption of region upstream to Mig1 caused some severe growth defects [14]. The resultant PfMig1^88^ ORF had stop codon at amino acid position 88 and hence the peptide of < 10 kD would end abruptly within the first zinc finger domain. PfMig1^88^ strain showed de-repressed cellulase and alcohol dehydrogenase expression. Slow hyphal growth phenotype and compact colony morphology in all CreA/Mig1 null alleles and deletion strains of filamentous fungi suggest that the absence of functional Mig1 might affect filamentation growth. To investigate this, we studied the growth of mutant and parent strains on both solid and liquid media. When we compared the growth by spotting on agar plates, we observed distinct difference in colony morphology, hyphal growth, and conidiation pattern between the parent strain NCIM1228 and PfMig1^88^. PfMig1^88^ strain showed compact colony size, reduced aerial hyphae, and visibly reduced growth on agar as observed for CreA null alleles and CreA deletion mutant of *A. nidulans* [14, 23]. However, when we examined the mycelial mass accumulation in liquid media, we found 30% more mycelial mass accumulation in PfMig1^88^ than the parent strain NCIM1228. Earlier studies on filamentous fungi reported equivalent mycelial mass accumulation in the presence and absence of catabolite repressor CreA in *N. crassa* [29] and *A. nidulans* [33]. We reasoned that if the mycelial mass accumulation is more for PfMig1^88^, its consumption of sugar would also be comparatively higher than the parent strain. We indeed found this to be true when consumption of glucose and cellobiose was measured for the two strains. Studies on *T. reesei* reported that no glucose was detected in extracellular media having cellobiose hydrolysis; however, we found accumulation of glucose until cellobiose was fully utilized [34]. This suggests that filamentous fungi have different evolving mechanisms and *P. funiculosum* NCIM1228 has evolved to grow on cellulosic biomass with the rate of hydrolysis of cellulosic disaccharide faster than the rate of hydrolyzed glucose uptake. At the same time, we observed 1.75-fold higher glucose uptake rate for PfMig1^88^ mutant as compared to NCIM1228, consequentially leading to utilization of both cellobiose and glucose equally well, which was contrary to the low specific growth rate and glucose consumption observed for *Aspergillus nidulans CreA* deletion mutant [33]. However, in *Talaromyces cellulolyticus*, *CreA* deletion mutant was found to have equivalent glucose consumption as the wild-type strain [19]. This suggests that functionality of Mig1 orthologs may vary among filamentous fungi. We next wanted to find out the reason for visibly slow growth on solid agar and for this we observed colony and hyphal morphology by microscopy. When we observed outer layer of NCIM1228 colony under microscope, we found parallel stacked structures of unbranched hyphae extending from core of the colony to the periphery and the rest of the terminal hyphae were arranged around these parallel structures. However, these parallel structures were not observed in the case of PfMig1^88^ at the periphery of the colony. They were found to be in the middle of the colony, with reduced length and frequency and never reached to the periphery of the PfMig1^88^ colony. We also observed altered pattern of the terminal hyphae of the two strains. While most of the terminal hyphae were unbranched in NCIM1228, the majority was found to be branched in PfMig1^88^ strain. Excessive branching in PfMig1^88^ might hinder the formation of parallel hyphal structures leading to compact, unorganized, and rough colonies. The absence of parallel structures might also compromise the invading capacity of PfMig1^88^. A very recent investigation in *S. cerevisiae* has found that Mig1 regulates filamentation pathway in glucose limiting condition by interacting with proteins of filamentation MAPK pathway at various levels [13]. Similar function of Mig1 might be possible in filamentous fungi as well where it might play an important role in filamentation growth. Enzyme secretion in filamentous fungi is known to happen at the hyphal tip; therefore, profuse lateral branching in the absence of Mig1 may also be one of the reasons for increased enzyme secretion in addition to the removal of catabolite repression.

### Mig1 regulates basal level as well as induction level of cellulase expression in *P. funiculosum*

Four major classes of enzymes, i.e., cellobiohydrolases or exocellulases, endoglucanases, β-glucosidases and xylanases, are primarily needed to hydrolyse cellulosic biomass. We examined the effect of Mig1^88^ null allele on the expression of all major classes of cellulases. We found sevenfold increased exocellulase and endocellulase activities in PfMig1^88^ secretome when compared with NCIM1228 secretome under complete repressing conditions (only glucose). This indicated high basal level expression of cellulases in the absence of Mig1. Further, twofold increased exocellulase and endocellulase activities found in PfMig1^88^ under complete de-repressing conditions (only Avicel) indicated prolonged induction of cellulase expression. This was further evident when increasing concentration of glucose in the presence of inducer had marginal impact on the expression of cellulases in PfMig1^88^ strain, but had major impact in the case of NCIM1228. However, we did not find substantial increase in β-glucosidase or xylanase activity in PfMig1^88^ strain. These results were further verified at the transcriptional level.

The growth and secretome profiles of PfMig1^88^ strain was further compared with parent strain in the production medium designed to support fast growth and high cellulase/hemicellulase production. We observed higher growth rate and twofold higher enzyme production for PfMig1^88^ strain, with maximum protein concentration reaching to > 14 g/l in its secretome. We believe our work here opens up further opportunities to improve the enzyme titer of the *P. funiculosum* Mig1^88^ strain via various genetic and bioprocess strategies.

## Methods

### Phylogenetic tree construction for Mig1 orthologs across fungal kingdom

All Mig1 CDS sequences were downloaded from Genbank (https://www.ncbi.nlm.nih.gov/genbank/). CDS sequence identities were generated using the pairwise multiple sequence comparison by EMBL-EBL log-expectation (MUSCLE) software. Multiple alignments of all the sequences were performed using the default parameters of MUSCLE for phylogenetic analysis. For drawing final conclusions, 41 organisms representing 31 Orders across all the fungi of industrial and scientific importance were taken for phylogenetic analysis. Phylogenetic trees were constructed using a neighbor-joining method and with 1000 bootstrap replicates, using the PHYPLIP v3.965 and visualized using iTOL—interactive Tree of Life [35]. Sequence alignment of Mig1 CDS region from 349 to 449 bp of representative fungal species was done by ClustalW and visualized using Jalview software.

### Cloning and plasmid construction

The gene and protein sequence of CreA of *T. cellulolyticus* were used for BLAST searches against the annotated *P. funiculosum* NCIM1228 genome sequence. A single open reading frame with high sequence homology was identified. The gene coding for Mig1, subsequently named PfMig1, spans 1248 bp with no introns. Primers P1 and P2 (see Additional file 1: Table S1) were used to amplify the coding region for Mig1 along with 500-bp upstream and 500-bp downstream region. The 2248-bp PCR product was cloned into pCambia1302 generating pCMig1. A PfMig1^88^ split marker deletion construct, named pCMig1^88^, was generated by removing 645-bp region of ORF of PfMig1 from pCMig1 by restriction digestion and replacing it with 1424-bp bleomycin selectable marker cassette at *Xba*I/*Ahd*I restriction site.

### Fungal strains and transformation

*Penicillium funiculosum* NCIM1228 was used as the background for constructing the mutant strain PfMig1^88^. PfMig1^88^ was constructed by transforming NCIM1228 with pCMig1^88^ via agrobacterium-mediated transformation. Transformants were selected for zeocin resistance and deletion was confirmed by amplification of newly acquired 3035-bp PfMig1^88^ split marker disruption construct in place of 2248-bp *mig1* coding region with flanking 5′ and 3′ region. Replacement of native *mig1* gene with PfMig1^88^ allele was also confirmed by RACE experiments using primers listed in Additional file 1: Table S1. For this, PfMig1^88^-positive transformant and NCIM1228 were grown in potato dextrose broth for 3 days and total RNA was isolated by using Qiagen RNeasy Mini Kit as per manufacturer’s instructions. cDNA from total RNA was synthesized using Thermo Fisher Scientific SuperScript III First-Strand Synthesis System as per manufacturer’s instructions. cDNA corresponding to full-length 1248-bp Mig1 transcript was amplified using primers P3 and P4 (Additional file 1: Table S1) [36]. For 5′-RACE of Mig1 transcript, 253-bp sequence from 5′-end of Mig1 transcript was amplified using primers P3 and P5. For 3′-RACE of Mig1 transcript, 319-bp sequence from 3′-end of Mig1 transcript was amplified using primers P4 and P6.

### Culture conditions

For all experiments, 50 ml of PD broth inoculated with 10^7^ conidiospores/ml was cultured for 24 h and was used as primary inoculum to inoculate 10% in secondary media. For comparing growth on agar plates, *P. funiculosum* NCIM1228 was grown at 30 °C in SC agar (0.67% yeast nitrogen base and 2% bacto-agar) plate supplemented with 2% carbon sources (unless otherwise stated) and incubated for 2, 7, and 14 days depending upon the type of carbon source used. To determine dry weight of mycelial biomass, the NCIM1228 and PfMig1^88^ strains were grown in SC medium containing 1% glucose or 1% cellobiose as sole carbon sources at 30 °C for 24 h with continuous shaking at 120 rpm. After 24 h, the mycelia were separated from the broth by filtration using Miracloth and the mycelia mass was estimated after drying at 70 °C until a constant weight was obtained. For studying colony characteristics and sporulation, LMP (1% malt extract and 0.05% soyapeptone) with 1.5% bacto-agar was used. For determining individual enzyme activities and transcript levels, NCIM228 and PfMig1^88^ were grown in modified Mandel medium [36] having different concentrations of glucose and Avicel. RCM medium containing soya peptone (24 g/l), KH_2_PO_4_ (5.9 g/l) (NH_4_)_2_SO_4_ (3.12 g/l), CaCl_2_·2H_2_O (0.05 g/l), yeast extract (0.05 g/l), wheat bran (24 g/l), and Avicel (21.4 g/l) was used as cellulase production media with final pH adjusted to 5.5. The flasks were incubated at 30 °C for 6 days with orbital shaking at 150 rpm (Innova 44, Eppendorf AG, Germany).

### Glucose and cellobiose uptake assay

To measure glucose and cellobiose utilization by NCIM1228 and PfMig1^88^ strains, equal number of conidiospores of NCIM1228 and PfMig1^88^ were initially grown in potato dextrose broth for 24 h. 10% of primary culture was used to inoculate 50 ml of SC media containing 1% glucose or 1% cellobiose. Samples were collected at different time intervals (0, 6, 12, 16, 18, 20, 22, and 24 h) and centrifuged, and supernatants were separated. Residual glucose and cellobiose in the supernatant were assayed using HPLC system (Agilent Technologies, USA) equipped with Aminex HPX-87H anion exchange column (Bio-Rad, USA) and a refractive index detector. Standards of glucose and cellobiose at 1 g/l were processed for HPLC and areas obtained were used to calculate residual glucose and cellobiose concentrations in the test samples.

### Protein estimation, SDS-PAGE, and Western blotting

Secretomes and mycelia obtained from the fungal culture were used for estimation of protein. Secretome was obtained by centrifuging the culture at 8000 rpm for 10 min, followed by buffer exchange using citrate–phosphate buffer (pH 4.0) with the help of 3 KD cut-off membrane. The sample was used to estimate the protein by BCA (Bicinchoninic acid) as well as to visualize protein bands on 10% SDS-PAGE (sodium dodecyl sulfate–polyacrylamide gel electrophoresis) gel. The mycelia harvested from the fungal culture were used to extract protein by alkaline treatment and protein was estimated using BCA method with bovine serum albumin as a standard. CBHI expression in the secretome of NCIM1228 and PfMig1^88^ was detected by Western blotting using rabbit anti-CBHI polyclonal antibodies [30] and mouse anti-rabbit HRP-conjugated secondary antibody (Cell Signaling Technology).

### Enzyme activity assays

All enzymatic activities were measured in citrate–phosphate buffer (50 mM, pH 4.0) at 50 °C. The activities of enzymes toward 0.5% Avicel, 1% CMC, and 1% beechwood xylan were measured using the dinitrosalicylic acid (DNSA) method. 200 μl of crude secretome was mixed with 200 μl of substrates and incubated for 30 min for CMC and beechwood xylan and 2 h for Avicel. The reaction was terminated by the addition of 400 μl of DNSA reagent and boiled for 10 min. The absorbance at 540 nm was measured relative to glucose or xylose standard curve. One unit of enzyme activity is defined as the amount of enzyme releasing 1 μmol of reducing sugar per min. β-glucosidase was assayed by determining the release of *p*-nitrophenol from *p*-nitrophenyl-β-d-glucopyranoside (pNPG). For this, 100 μl of crude secretome was mixed with 100 μl of substrate (1 mM) and incubated for 30 min. The reaction was stopped by adding 200 μl of 1 M sodium carbonate (pH 11.5), and the release of 4-nitrophenol was quantified at 410 nm using a 4-nitrophenol standard curve. One unit of enzyme activity was defined as the amount of protein that released 1 μmol of *p*-nitrophenol per min. FPA (filter paper assay) was conducted to assess total cellulase activity of secretome produced in production media as mentioned by [36]. The assay requires fixed degree of conversion of substrate, i.e., a fixed amount (2 mg) of glucose released from 50 mg of filter paper within 60 min at 50 °C. Total cellulase activity is described in terms of “filter paper units” (FPU) per milliliter of original (undiluted) enzyme solution.

### Expression analysis via real-time PCR

For real-time PCR experiments, mycelia were harvested by filtration and lyophilized in liquid nitrogen. RNA was extracted using RNeasy kit (Qiagen) according to the manufacturer’s instructions. RNA was DNase treated (Invitrogen) prior to cDNA synthesis. 100 ng of RNA was used as template in each quantitative real-time PCR (qRT-PCR) reaction. cDNA synthesis control was performed to ensure the absence of DNA contamination. qRT-PCR was carried out using iTaq™ Universal SYBR^®^ Green Supermix (Bio-Rad) and Bio-Rad CFX96 qPCR detection system. Primers for test and control transcripts were designed using boundary sequence of two exons to avoid any amplification from genomic DNA contamination (Additional file 1: Table S1). qRT-PCR was done in biological triplicates with actin and tubulin as the endogenous control. Relative expression levels were normalized to tubulin, and fold changes in RNA level were the ratios of the relative expression level of PfMig1^88^ to NCIM1228 under repressing conditions and cellulase inducing conditions to no carbon conditions [37].

### Microscopy

For conidia germination experiment, 10^6^ spores were inoculated in 50 ml of SC media for 18 h. Percentage germination was determined microscopically by counting the numbers of germinating conidia (conidia with a visible germ tube) in a population of more than 100 in three independent experiments.

For observing colony structures, spores were spotted on thin LMP agar plates and colony morphology was observed after 48 h of incubation. For microscopy, plates were flooded with lactophenol blue and transverse sections were cut and observed under 4×, 20×, and 60× magnifications. For counting branching pattern in terminal hyphae, more than 100 hyphae were counted in three independent experimental setups. For conidia observation, colonies were allowed to grow for 7 days on LMP agar and conidia were observed under microscope.

## Additional file

**Additional file 1: Figure S1.** Phylogenetic tree of protein sequence of all reported Mig1 homologs across fungal kingdom. **Figure S2.** Sequence alignment of zinc finger domains of Mig1 homologs from industrially relevant filamentous fungi. **Figure S3.** Sanger sequencing chromatogram showing transversion (highlighted in blue) at 400th nucleotide position in Mig1 gene in *P. funiculosum* NCIM1228.** Figure S4.** Nucleotide sequence of Mig1 gene of *P. funiculosum* NCIM1228. **Figure S5.** Alignment of PfMig1 ORF (from 349 to 449 bp) with corresponding region of Mig1 of 29 closely related fungal isolates to check the presence of internal stop codon in these isolates. **Figure S6.** Schematically representation of various branching patterns of *P. funiculosum* seen under microscope. **Table S1.** List of Primers used in the study.

## Abbreviations

NCIM1228: *P. funiculosum* NCIM1228
Mig1^134^: native allele of *P. funiculosum* NCIM1228
Mig1^88^: null allele of Mig1 in *P. funiculosum* NCIM1228
PfMig1^88^: Mig1-disrupted strain of *P. funiculosum* NCIM1228
FPU: filter paper unit
CBH: cellobiohydrolase
EG: endoglucanase
BG: beta-glucosidase
Xyl: xylanase
2-DG: 2-deoxyglucose
AA: allyl alcohol
