# Supplementary material for: Disruption of zinc finger DNA binding domain in catabolite repressor Mig1 increases growth rate, hyphal branching, and cellulase expression in hypercellulolytic fungus Penicillium funiculosum NCIM1228
Source: Biotechnol Biofuels. 2018 Jan 25;11:15. doi: 10.1186/s13068-018-1011-5 (PMC5784589; doi:10.1186/s13068-018-1011-5)
Supplement: Supplementary file 1 — Additional file 1: Figure S1. Phylogenetic tree of protein sequence of all reported Mig1 homologs across fungal kingdom. Figure S2. Sequence alignment of zinc finger domains of Mig1 homologs from industrially relevant filamentous fungi. Figure S3. Sanger sequencing chromatogram showing transversion (highlighted in blue) at 400th nucleotide position in Mig1 gene in P. funiculosum NCIM1228. Figure S4. Nucleotide sequence of Mig1 gene of P. funiculosum NCIM1228. Figure S5. Alignment of PfMig1 ORF (from 349 to 449 bp) with corresponding region of Mig1 of 29 closely related fungal isolates to check the presence of internal stop codon in these isolates. Figure S6. Schematically representation of various branching patterns of P. funiculosum seen under microscope. Table S1. List of Primers used in the study. [file 13068_2018_1011_MOESM1_ESM.docx]

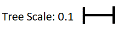

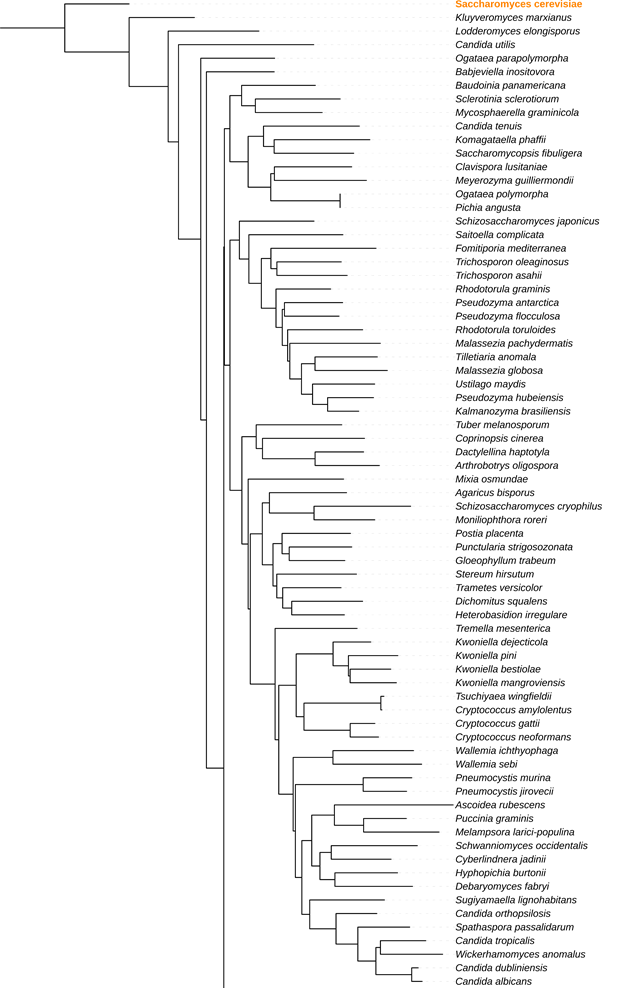


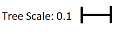

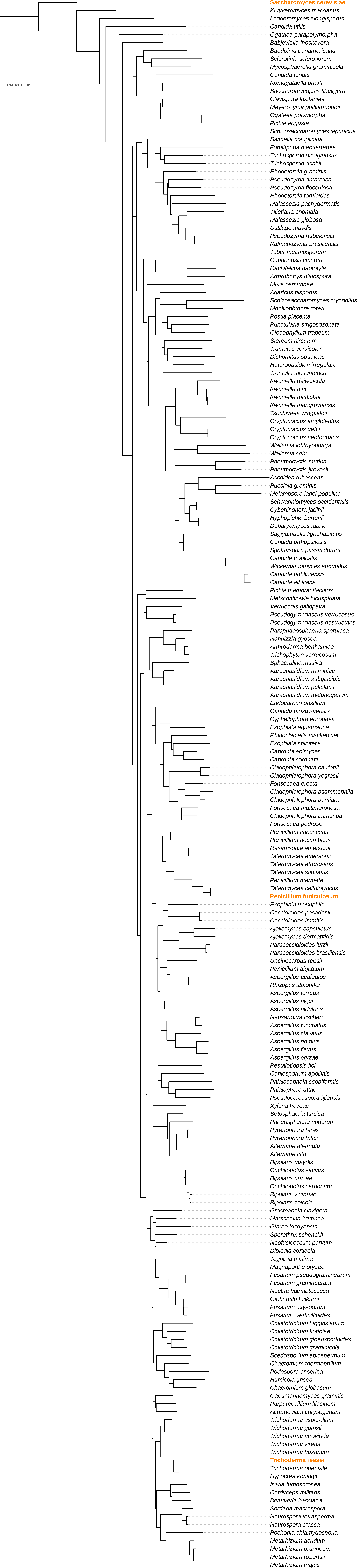


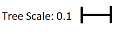

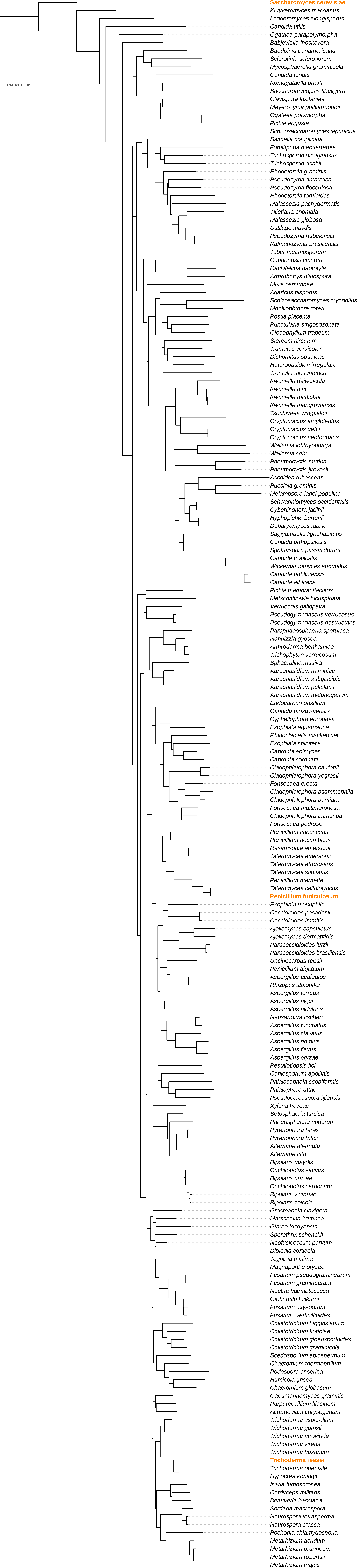


**Figure S1.** Phylogenetic tree of protein sequence of all reported Mig1 homologs across fungal kingdom.


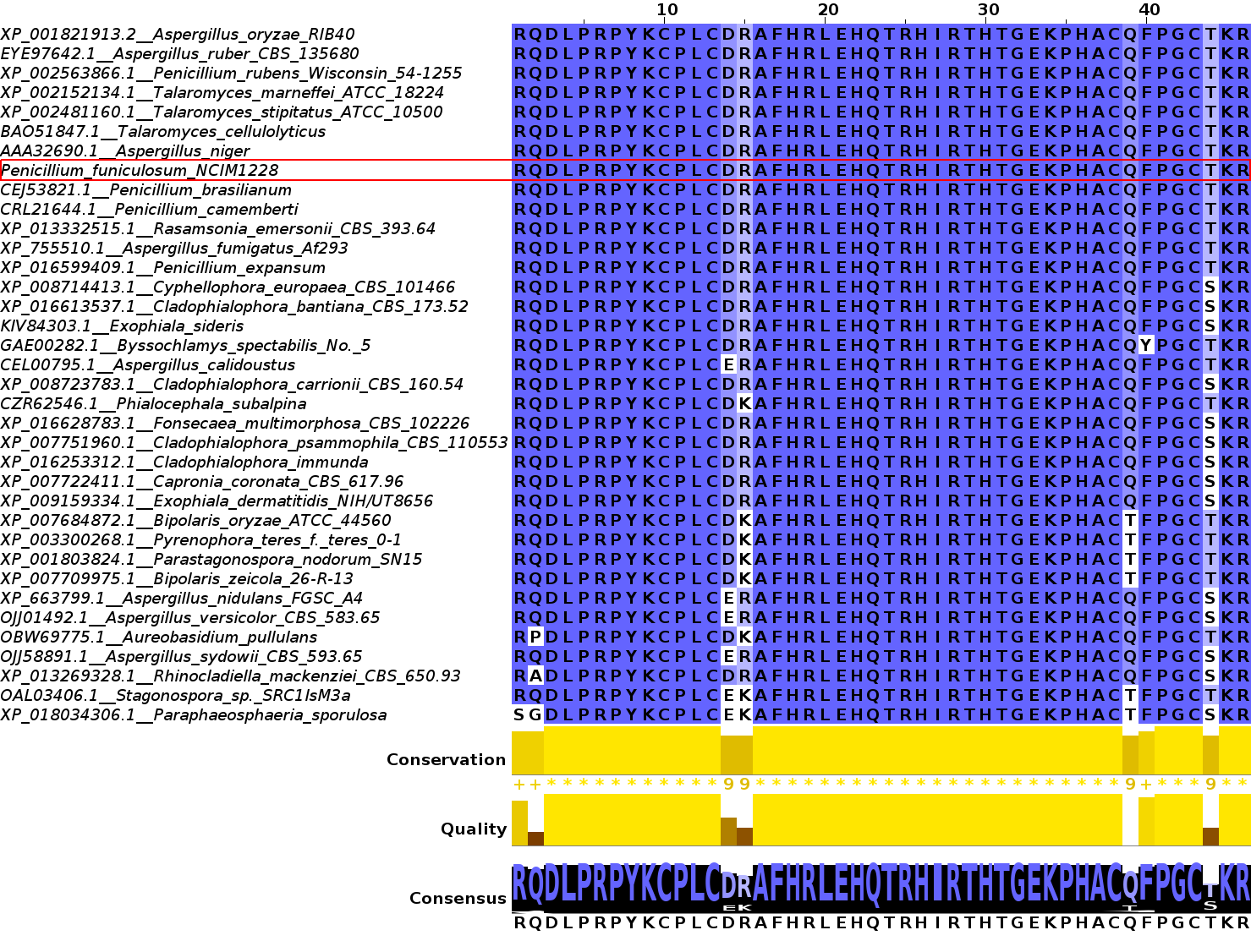


**Figure S2.**  Sequence alignment of zinc finger domains of Mig1 homologs from industrially relevant filamentous fungi. The zinc finger module region was identified by using Interproscan tool [1]. The identified regions were extracted from CreA protein and proteins of other listed species, which were aligned by using Clustal Omega [2] and the alignment was visualized by using Jalview tool [3]. Zinc finger domains were found to be highly conserved among Mig1 homologs, showing variability at only 7 positions in 46 amino acid sequence stretch.


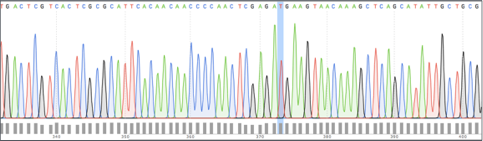


**Figure S3.** Sanger sequencing chromatogram showing transversion (highlighted in blue) at 400th nucleotide position in Mig1 gene in *P. funiculosum* NCIM1228. The gene for Mig1 cloned in pMig1 construct was sequenced commercially using Sanger sequencing method at Macrogen Inc. and chromatogram for the region having point mutation leading to stop codon has been shown.

ATGTCACCGTCATCTTCGTCAGTGGGTTTTTCCAATCTGCTGAACCCACAGTCAGACTCTGTCGAGTCTACGGATAACACATCTTCACCTGCTACCACCACTACCACCGGCACAGACTCCAACTCAGACAAGGAAATGGCGTCCTCTGTCAGTCTGCTCCCACCGCTCATGAAAGGTGCCCGCCCCGCCGCGGAGGAAGTGCGACAGGATCTCCCTCGTCCATACAAGTGTCCTCTTTGCGATCGTGCTTTCCATCGTCTAGAGCACCAAACTCGTCACATTCGTACTCACACCGGCGAGAAACCCCATGCCTGCCAGTTTCCAGGCTGCACGAAACGGTTCAGTCGTTCAGATGAATTGACTCGTCACTCGCGCATTCACAACAACCCCAACTCGAGATGAAGTAACAAAGCTCAGCATATTGCTGCGGCTGCGGCCGCCGGTCAGGATTCGGGTATGCTTAACGCTGCTGCCTCGATGATGCCTCCTCCAAGCAAACCCATTACTCGCTCGGCTCCAGTGTCTCAGGTCGGATCTCCGGATGTGTCTCCTCCGCACTCTTACACCAACTACACCTCGCATTTGCGGGCGGGTCTGGGTCCTTATTCACGCAACAGCGACCGTGCTTCATCTGGTATGGATATTAATTTGCTCGCGACTGCTGCTTCACAAGTCGAGCGCGATCACTACGGAGGCTCGTCTCGTCATTACCCTTTCAGCTCTCGATACTCGGGTACTCCTGGACGTCTGCCGTCGCTTTCCGCCTATGCCATTTCTCAGAGCATGAGCCGGTCGCATTCTCACGAGGATGAGGACAACTACGGACATCACCGGGTTAAGCGCTCTCGTCCTAATTCACCAAACTCGACTGCGCCATCCTCGCCTACCTTCTCTCACGACTCATTGTCGCCTACCCCCGACCACACTCCTCTCGCAACCCCAGCACACTCGCCTCGTTTGCGTCCTTATGGAGCCGCAGATTTGCAATTACCTTCCATCCGTCATTTGTCGTTACACCACACTCCCGCACTCGCACCAATGGAGCCTCAAGCCGAGGGACCTAATGTTTACAACCCCGGTCAGCACCACGGTGGACCCAGCATCACGGACATCATGAGCAGGCCCGACGGCACCCAGCGTAAACTTCCTGTTCCGCAAGTACCCAAAATCCCGGTGCAGGACATGTTGGCACCGAACGGATATTCCTCCAACACTCCGTCCGTCAACGGTTCCGTGATGGAGTTATAA

**Figure S4.** Nucleotide sequence of Mig1 gene of *P. funiculosum* NCIM1228. The mutation at 400^th^ position leading to stop codon is denoted in red.


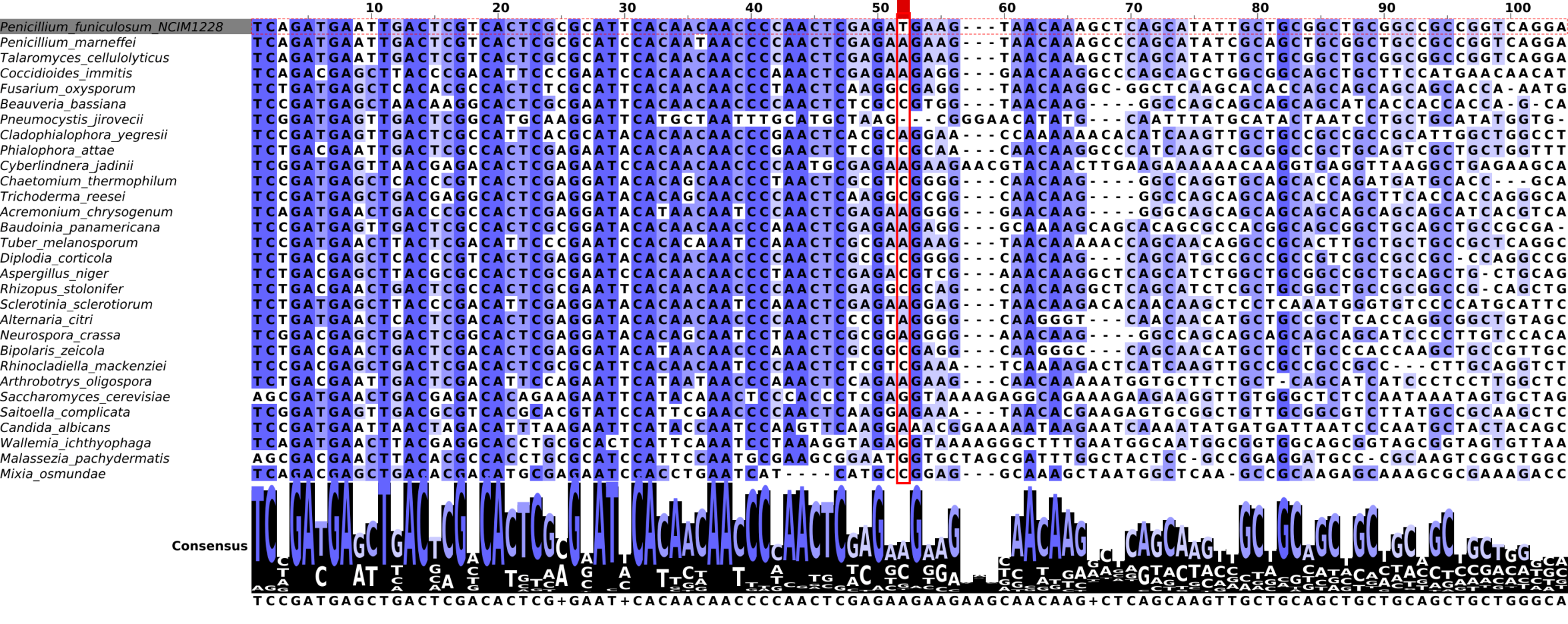


**Figure S5**. Alignment of PfMig1 ORF (from 349 to 449 bp) with corresponding region of Mig1 of 29 closely related fungal isolates to check the presence of internal stop codon in these isolates. Red box represents the nucleotide position at which the transversion (possibly from A to T) in PfMig1 was observed which led to generation of a stop codon.

**
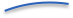
% Unbranched**

**
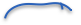
**

**% Nicked bifurcation**

**
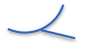
**

**% Bifurcation**

**
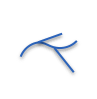
**

**
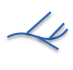
% Trifurcation**

**% Quadrafurcation**

**Figure S6.** Schematically representation of various branching patterns of *P. funiculosum* seen under microscope. Statistics of the branching patterns have been presented in Table 1.

**Table S1.** List of Primers used in the study

| Experiment | Primer |
| --- | --- |
| Mig1^88^ cassette construction | P1 (5′-GACACCGCGCGCGTGAGATACGTATATACGCAGCTACCAATC-3′)  P2 (5′-GAGAAACTCGAGCTTCCCCCACACGGTACTCC-3′) |
| RACE Experiments | P3 (5’-ATGTCACCGTCATCTTCGTCAG-3’)  P4 (5’-CTCCATCACGGAACCGTTGAC-3’).  P5 (5’-GGAAAGCACGATCGCAAAGAG-3’)  P6 (5’- CACTCCTCTCGCAACCCCAG-3’) |
| RT-PCR Primers | CBHI F (5’-GCAAACACGAAGCTGGTATGG-3’)  CBHI R (5’-GGTGACATAGGAGCTGCCGG-3’)  CBHII F (5’-CTGGGGACAATGTGGCGG-3’)  CBHII R (5’-GTTGTCGGCGGCGATGTC-3’)  EG-GH5 F (5’-GCAACCATTGGTGAATTCATCAGTCAG-3’)  EG-GH5 R (5’-CTGCCATTGTACCTTCCATAATTGTGAG-3’)  EndoGH45 F (5’-GATTCCTGGCAGCTCGGC-3’)  Endo GH45 R (5’-AGGTTGGTGACCATAACGATGATG-3’)  Xyl(GH11-CBMI) F (5’-GTGCTGCTCGCTCCATTACCTAC-3’)  Xyl(GH11-CBMI) R (5’-CTTGGTAGGTGCCACCGTCAG-3’)  Xyl (GH10-CBMI) F (5’-GCAATGAAATGGCAACCCACCG-3’)  Xyl (GH10-CBMI) R (5’-CCTTCAAGGCAGCAATAAGGGTTG-3’)  Xyl-GH11 F (5’-GGTGATGCTAACCCCATCACCTAC-3’)  Xyl-GH11 R (5’-AGATATCATAGGTGCCGCCGTC-3’)  BG-GH3 F (5’-CTGGCGAAGGGTACATAACAGTCG-3’)  BG-GH3 R (5’-GCCAGCCCATACTACGGC-3’)  BG-GH1 F (5’-CAATGACACTGTTAGATATGCCATCTCAG-3’)  BG-GH1 R (5’-GACCACGCGGCTTGACC-3’)  Actin F (5’-CATTGTCATGTCTGGCGGTACTAC-3’)  Actin R (5’-CGTACTCCTGCTTGGAGACCC-3’)  Tubuin F (5’-ATTGCTCAGGTTGTCTCCTCCATC-3’)  Tubulin R (5’-ATTGCTCAGGTTGTCTCCTCCATC-3’)  Intron (-ve control)  intron F (5’-GTCAGTGGCCAAACATCACTACTG-3’)  intron R (5’-GGATGACAATCCATGGAGGTCGTATG-3’) |

**Supplementary References**

1. Zdobnov, E. M., & Apweiler, R. (2001). InterProScan–an integration platform for the signature-recognition methods in InterPro. *Bioinformatics*, *17*(9), 847-848.
2. Sievers, Fabian, et al. "Fast, scalable generation of high‐quality protein multiple sequence alignments using Clustal Omega." *Molecular systems biology* 7.1 (2011): 539.
3. Waterhouse, Andrew M., et al. "Jalview Version 2—a multiple sequence alignment editor and analysis workbench." *Bioinformatics* 25.9 (2009): 1189-1191.
